# Conserved protein folds underpin the diversification of secreted proteins in a fungal pathogen

**DOI:** 10.1186/s12866-026-05518-2

**Published:** 2026-08-29

**Authors:** Thaís C. S. Dal’Sasso, Eva H. Stukenbrock

**Affiliations:** 1 Environmental Genomics, Christian-Albrechts University of Kiel, Am Botanischen Garten 9, Kiel, 24118 Germany; 2 Max Planck Institute for Evolutionary Biology, August-Thienemann-Str. 2, Plön, 24306 Germany

**Keywords:** Protein structure and evolution, Effectors, Antimicrobial proteins, Network analysis, *Zymoseptoria passerinii*

## Abstract

**Background:**

During host colonization, fungal plant pathogens secrete effector-like proteins that alter host cell physiology and target plant-associated microbes. However, rapid evolution and low sequence conservation hinder the study and characterization of these proteins. The fungus *Zymoseptoria passerinii* infects *Hordeum* spp. and includes lineages adapted to wild and domesticated barley. To date, the evolution of effector-like proteins in this species has not been addressed.

**Results:**

We combined multiple structure-based and network analyses to unravel the secretome of *Z. passerinii*. We first compared AlphaFold2 and ESMFold predictions to establish the baseline for structural analyses. We identified 72 structural clusters in the secretome, revealing fold-level relationships across divergent sequences. We showed that effector-like proteins with predicted host immune-interfering functions evolved from a limited group of protein folds, whereas proteins with predicted antimicrobial properties were distributed across fold groups. Physicochemical comparisons indicate that putative antimicrobial effectors predominantly emerged through amino acid replacements on common effector-enriched scaffolds in *Z. passerinii*, reconfiguring surface charge and electrostatics. We analyzed intra- and interspecific variation in selected effector-enriched families by comparing *Z. passerinii* proteins and homologs across the genus *Zymoseptoria*. We describe constrained core folds, with local variation in loop and surface-exposed regions, consistent with fold stability while still enabling protein diversification. We further report that putative antimicrobial effector homologs are broadly distributed across the genus despite sequence divergence.

**Conclusions:**

The secretome of *Z. passerinii* is organized around common structural folds that support diverse biological roles, including host manipulation and host-associated microbial interactions. Conserved scaffolds combined with surface and physicochemical variation likely contribute to rapid adaptive evolution of effector-like proteins in *Z. passerinii.*

**Supplementary Information:**

The online version contains supplementary material available at https://doi.org/10.1186/s12866-026-05518-2.

## Background

Eukaryotic microorganisms use secreted proteins as molecular mediators of their interactions with the environment. Microbial secretomes can participate in nutrient acquisition through enzymatic degradation of substrates, modulate biotic and abiotic stress responses, promote interactions with other organisms, and contribute to growth in diverse habitats [1–4]. Secretome repertoires of pathogens frequently include rapidly evolving proteins that adapt in response to selection pressures imposed by hosts and associated microbes [4]. As a result, secretomes are shaped by gene family dynamics, duplication events, and adaptive evolution linked to ecological changes and niche specialization [5, 6]. Thus, secretome repertoires can be surveyed to study microbial adaptation; however, extensive genetic variation and low sequence conservation in a majority of secreted fungal proteins challenge the inference of homologies and functions.

In plant-associated fungi, effectors are secreted proteins that play important roles in pathogenesis as they can interfere with host defenses at multiple levels, shaping the outcome of host infection [1, 7, 8]. Hereafter, we refer to predicted effector candidates as effector-like proteins. Across pathosystems, fungal effectors can alter host cells by suppressing pattern-triggered immunity [9, 10], perturb reactive oxygen species signaling [11], alter hormone balance [12], or interact with host components to evade recognition [13, 14]. More recent works have documented that some fungal effectors have antimicrobial properties [15]. Such antimicrobial effectors are increasingly recognized as components of plant infection biology as they can inhibit microbial competitors in host tissues or modulate the recruitment of host-associated microbes, thereby reshaping the host niche in favor of the pathogen [3, 15, 16]. Thus, antimicrobial effectors expand the functional space of effector-like proteins from host-targeting virulence factors to multifunctional molecules that act at the host–pathogen–microbe interface.

Despite their roles in virulence, most fungal effector-like proteins remain uncharacterized due to small size, rapid evolution, and lack of conserved sequence domains [4, 17]. For that reason, conventional sequence-based methods often fail to predict their functions as well as their evolutionary relationships among distantly related taxa [18, 19], limiting our understanding of their roles in host–microbe interactions. Nevertheless, many effector-like proteins can adopt analogous three-dimensional structures that are important for pathogenesis [6, 18, 20–22], even in the absence of detectable sequence similarity. These sequence-unrelated but structurally similar effectors [6] exhibit common folds associated with key biological roles during host infection [22]. Therefore, structure-based approaches can be used to uncover protein homologies and similarities not recognized from sequence comparisons, and thereby shed light on mechanisms of fungal pathogenicity that would otherwise remain cryptic among dynamic and fast evolving proteins [6, 18, 22, 23].

The latest developments in protein structure prediction methods provide new opportunities for investigating the effector biology of fungal plant pathogens [22]. Tools such as AlphaFold [24] and ESMFold [25] have allowed the prediction of protein structures on a large scale across a wide range of organisms. These include the plant pathogenic fungi *Magnaporthe oryzae* [20], *Fusarium oxysporum* [26], *Ustilago maydis* [27], and *Venturia inaequalis* [28]. In this context, comparative structural genomics can be used to expand our understanding of effector evolution [6, 20]. Effector families share common ancestry evidenced by conserved protein folds [6]. These highly variable proteins are thought to have diverged through multiple duplication events followed by sequence divergence, sometimes resulting in neofunctionalization and compatibility with new target proteins [6, 22]. However, functional diversification and evolutionary changes at the structural level have so far been poorly explored for secreted proteins of pathogenic fungi.

The fungal genus *Zymoseptoria* includes several grass-infecting species [29]. The most studied species in the genus is the wheat pathogen *Z. tritici*, the causal agent of the Septoria tritici blotch disease [30, 31]. A close relative of *Z. tritici*,* Zymoseptoria passerinii* is the causal agent of Septoria speckled leaf blotch in barley [32]. Although *Z. passerinii* is relatively rare in agricultural production systems, the pathogen persists in natural ecosystems in the Middle East, where it infects species of the genus *Hordeum* [33, 34]. These populations of *Z. passerinii* overlap with the center of origin and diversity for Septoria pathogens and their grass hosts [35]. Notably, *Z. passerinii* comprises genetically distinct lineages that infect either wild or domesticated barley, suggesting a long co-evolutionary relationship with the grass hosts [34]. Previously, we characterized the putative effector repertoire in a *Z. passerinii* isolate associated with cultivated barley and further uncovered a large set of unique effectors without sequence homology to effectors predicted in other *Zymoseptoria* species [29]. Moreover, we showed that *Z. passerinii* can alter leaf microbiome composition and may engage in interactions with resident microbes [36]. Therefore, *Z. passerinii* likely deploys a diverse array of secreted proteins to manipulate host responses and antimicrobial effectors to mediate microbial manipulation during barley colonization. Thus, *Z. passerinii* serves as an interesting model to study ecological interactions and host specialization of fungal pathogens occurring on wild and cultivated plants [34].

Structure-based analyses offer a promising approach to study rapidly evolving proteins across eukaryotic microorganisms. In this study, we investigate the structural landscape of the secretome of a fungal pathogen, using *Z. passerinii* isolate Zpa796, sampled from the wild barley species *H. murinum* ssp. *glaucum* [34], as a model system. We selected this isolate as it represents a reference isolate of *Z. passerinii* populations on wild barley [34]. Zpa796 causes disease in wild barley, affects host-associated microbial communities, and has the highest-quality long-read genome assembly available for the species to date [34, 36]. Despite its ecological relevance, the repertoire of proteins that contribute to virulence or microbiota manipulation remains elusive for this pathogen. By extending secretome-wide structural analysis to *Z. passerinii*, we aim to identify candidate effector-associated folds that can guide future functional assays and expand our understanding of pathogen evolution in wild pathosystems. We hypothesize that structural features of fungal secreted proteins are tied to their biological roles in host colonization and microbial interactions, and that proteins sharing similar folds can act as evolutionary scaffolds supporting functional diversification. To establish a robust baseline for the structural analyses, we first compared the results obtained with AlphaFold2 (AF2) and ESMFold. Although both methods are widely used for protein modelling, they differ in computational cost and runtime [25], and their accuracy has not been systematically compared for fungal secretomes. We next conducted structural annotation and network-based clustering to identify common folds and uncover homology relationships and similarities within the secretome of *Z. passerinii*, and we sought to determine whether specific folds were associated with effector-like or putative antimicrobial proteins. We compared physicochemical properties to understand the distribution of antimicrobial activity across effector-like proteins and investigated how conserved structural scaffolds may allow functional diversification. Through an integrative approach, we show that the secretome of *Z. passerinii* is organized around a set of protein folds that support diverse biological roles, and that conserved cores combined with physicochemical variation contribute to effector evolution.

## Results

### Comparison of structural predictions from AlphaFold2 and ESMFold

**Fig. 1:**
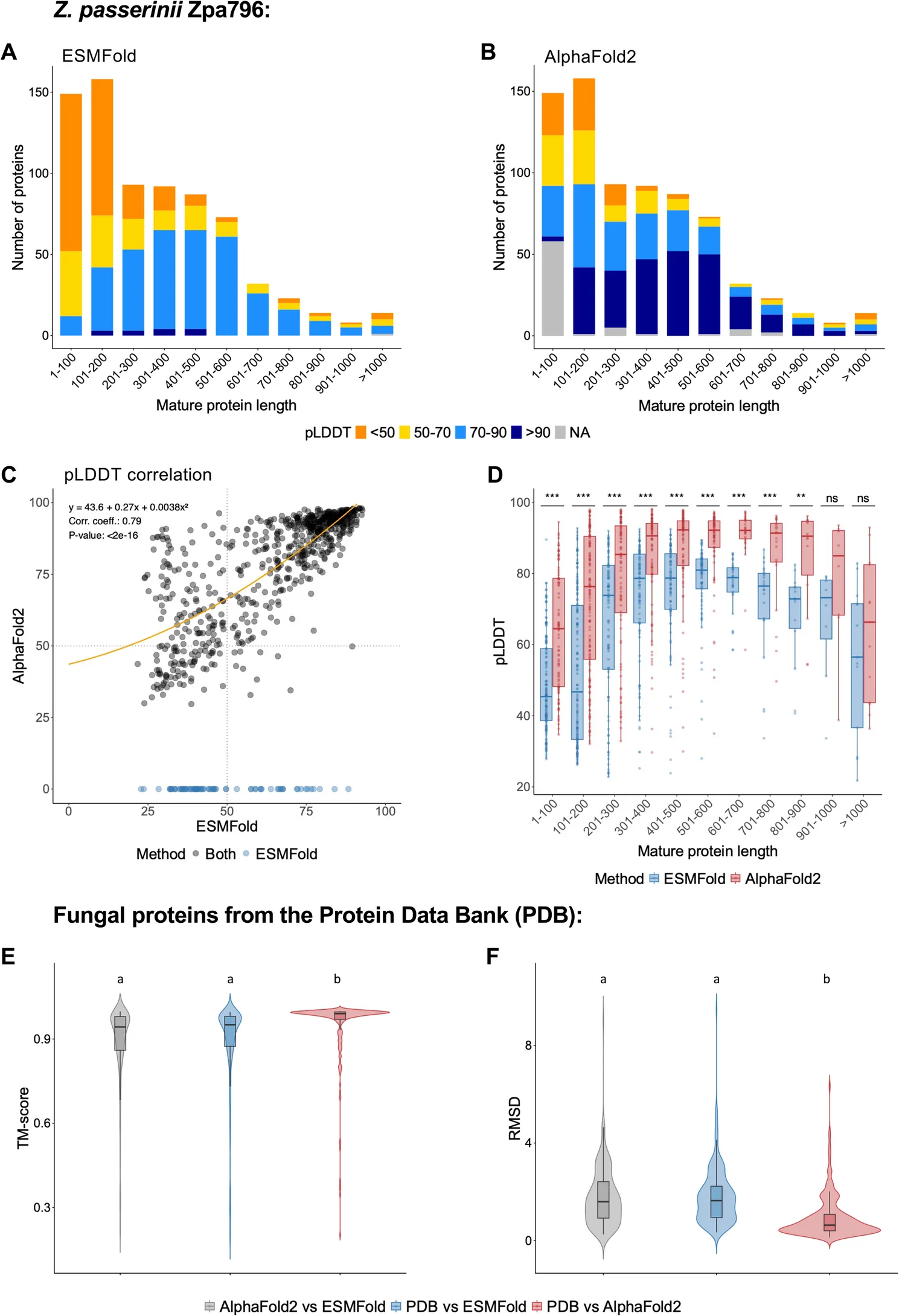
Comparison of protein structural predictions performed by ESMFold and AlphaFold2 (AF2). Protein structures were predicted for all secreted proteins of the fungal pathogen *Z. passerinii* (isolate Zpa796). Confidence scores of protein structures according to mature protein lengths (number of amino acid residues) are shown as the mean per-residue measure of local confidence (pLDDT) values as computed by (**A**) ESMFold and (**B**) AlphaFold2. Proteins from Zpa796 that failed the structural predictions are labeled as NA (grey). (**C**) Spearman’s correlation of pLDDT values for individual protein structures predicted by ESMFold and AF2 for the secretome of Zpa796; proteins uniquely predicted by ESMFold are shown in blue. (**D**) Differences in mean pLDDT values per classes of mature protein lengths in Zpa796; significance levels were assessed using the Wilcoxon test (** for p-value < 0.01, *** for p-value < 0.001). Mean structural similarity measured by (**E**) Template modeling (TM) score and (**F**) Root mean square deviation (RMSD) between fungal crystallized protein structures (retrieved from the Protein Data Bank, PDB) and their predicted structures by ESMFold and AF2

Due to the low sequence conservation and the absence of conserved protein domains, the functional relevance for most fungal secreted proteins is unknown. We therefore, firstly, set out to establish a robust pipeline for predicting the structures of *Z. passerinii* secreted proteins. We predicted a total of 743 secreted proteins in the proteome of *Z. passerinii* Zpa796 (Supplementary Material 1: Table S1) and used these for structure modeling with AF2 [24] and ESMFold [25]. We predicted a total of 670 protein structures using AF2 and 742 using ESMFold, corresponding to more than 90% of the secretome (Supplementary Material 1: Table S2). Out of 73 mature proteins that failed predictions with AF2, 58 were peptides shorter than 100 amino acids (Fig. 1A-B, Supplementary Material 1: Tables S1 and S2). Overall, AF2-predicted structures displayed significantly higher mean pLDDT values (predicted local distance difference test, p-value < 0.001) than those predicted by ESMFold (Fig. 1A-D, Supplementary Material 2: Fig. S1A). The mean pLDDT value for AF2-predicted structures was 78.23 compared to 63.03 for ESMFold-predicted structures. We note that on the 0–100 pLDDT scale, values above 70 indicate confidently modelled proteins, whereas values below 70 reflect lower confidence values [24, 25]. Despite the difference, pLDDT values from AF2 and ESMFold were positively correlated (*r* = 0.79, p-value < 0.01), although non-linear, across the secretome of *Z. passerinii* Zpa796 (Fig. 1C). To further understand the prediction confidence, we analyzed pLDDT values as a function of mature protein length and predicted disordered residues for the secretome of Zpa796 (Fig. 1D, Supplementary Material 2: Fig. S2). AF2 structures exhibited significantly higher mean pLDDT values (p-value < 0.01) than ESMFold for mature proteins up to 900 amino acids long. Structure prediction of short mature proteins (up to 100 residues) remains particularly challenging, but in cases where both software tools successfully predicted a structure, AF2 provided higher pLDDT values than ESMFold (Fig. 1D). Lower prediction confidence was also correlated with a higher proportion of predicted disordered residues for both AF2 and ESMFold models (Supplementary Material 2: Fig. S2), suggesting that low pLDDT values may partly reflect intrinsic flexibility or partial disorder among the secreted proteins.

We next assessed structural similarity between AF2 and ESMFold predictions for the secretome of *Z. passerinii* Zpa796 using two standard metrics, the template modelling (TM) score and the root mean square deviation (RMSD), calculated from pairwise alignments of mature proteins predicted by both methods (Supplementary Material 2: Fig. S1B-E, Supplementary Material 1: Table S2). The TM-score summarizes global fold similarity and RMSD reports the average distance between aligned Cα atoms, allowing us to test how similar the results from the two prediction methods were. Mature proteins shorter than 100 amino acids had the lowest TM-scores and highest RMSD values between methods, indicating larger structural discrepancies between AF2 and ESMFold models, whereas proteins of intermediate length (401–900 amino acids) showed higher TM-scores and lower RMSD values (Supplementary Material 2: Fig. S1D-E).

To validate whether the results we observed for *Z. passerinii* Zpa796 hold for other fungal proteins, we next benchmarked AF2 and ESMFold predictions against experimentally determined structures using a set of crystallized fungal proteins from the Protein Data Bank (PDB [37]) (Fig. 1E-F, Supplementary Material 2: Fig. S3). In total, we retrieved 228 crystallized protein structures and used their amino acid sequences as input for AF2 and ESMFold calculations (Supplementary Material 1: Table S3). AF2 successfully predicted structures for 226 proteins, only failing prediction for two small proteins from the PDB: 2ALC (a DNA-binding protein from *Aspergillus nidulans*) and 2HGO (the cassiicolin toxin from the plant pathogen *Corynespora cassiicola*). ESMFold successfully predicted structures for all 228 proteins. When we compared the prediction confidence of the two methods, AF2-predicted structures exhibited significantly higher mean pLDDT values (93.36) than those predicted by ESMFold (80.82) for fungal proteins retrieved from the PDB (p-value < 0.001). Next, we assessed the structural similarity between AF2 and ESMFold models relative to crystallized structures from the PDB (Fig. 1E-F, Supplementary Material 2: Fig. S3). AF2 predictions showed significantly higher mean TM-score and lower RMSD values when compared to PDB structures (mean TM-score = 0.95, mean RMSD = 0.93 Å; p-value < 0.01) (Fig. 1E-F). In particular, we found significantly higher TM-scores and lower RMSD values for PDB–AF2 alignments in proteins ranging from 101 to 700 amino acids long (Supplementary Material 1: Fig. S3). Together, these results reflect greater structural similarity between AF2 predictions and the crystallized fungal structures from PDB than ESMFold predictions compared to the same PDB proteins.

Thus, we conclude that AF2 outperformed ESMFold in terms of prediction confidence and structural accuracy for the fungal proteins that we analyzed here. We therefore used the 670 AF2-predicted structures for *Z. passerinii* for further downstream analyses.

### Structure-based annotation expands putative functions of the secretome

**Fig. 2:**
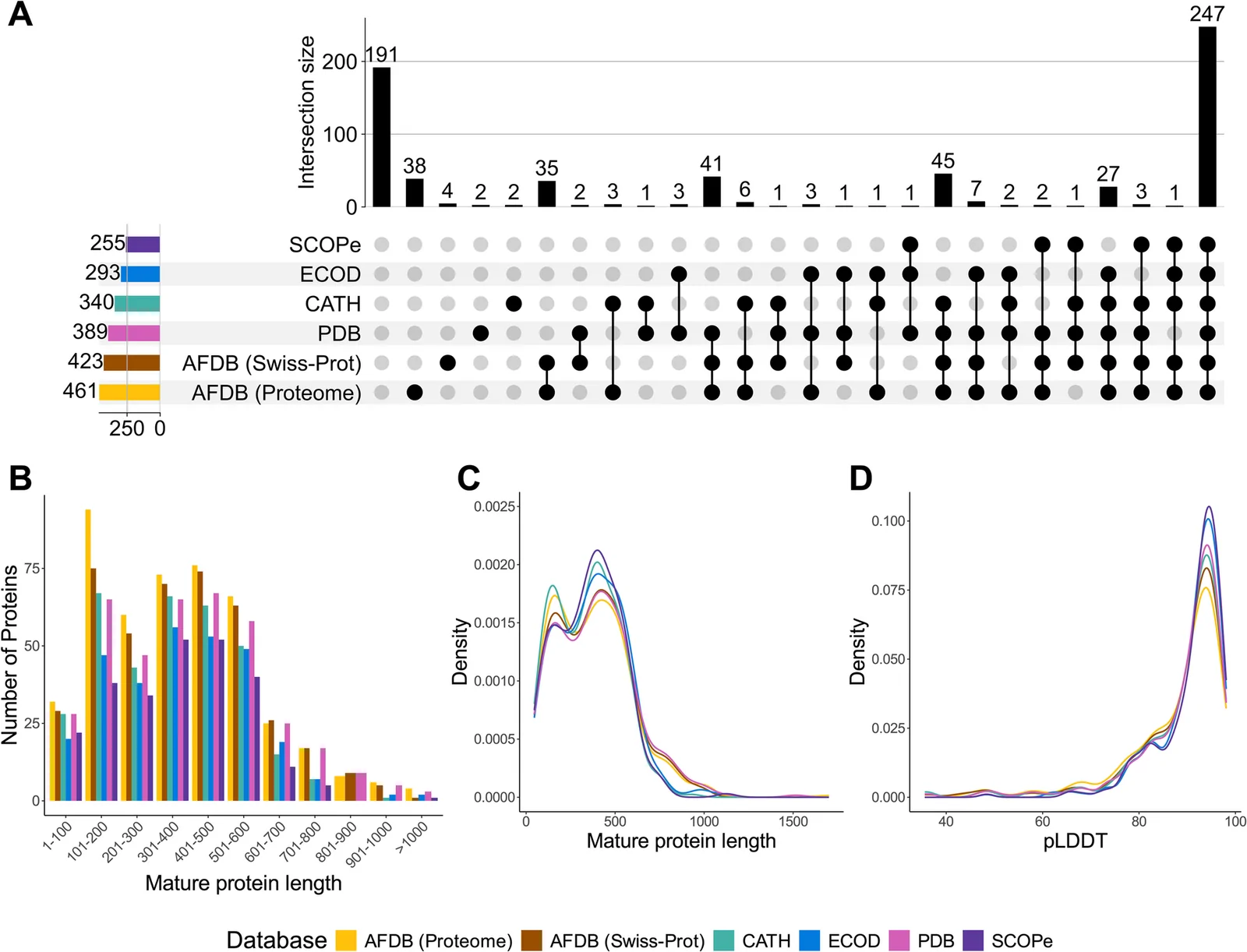
Structure-based annotation of 670 proteins from *Zymoseptoria passerinii* Zpa796. (**A**) The overlap of protein annotations across structural databases. (**B**) Number of proteins annotated by database according to categories of mature protein length. Distribution of protein structures annotated according to (**C**) Mature protein length and (**D**) pLDDT values across structural databases; AlphaFoldDB (AFDB) subsets “Proteome” and “Swiss-Prot”, CATH, ECOD, PDB and SCOPe

We annotated the secretome of *Z. passerinii* Zpa796 using amino acid sequences and AF2-predicted structures (Supplementary Material 2: Fig. S4A, Supplementary Material 1: Tables S1 and S4). Overall, 408 secreted proteins were annotated by sequence-based methods while 479 proteins were annotated using structure-based approaches. To define fold-level structural matches, we used a TM-score threshold of 0.5 and selected the best hit based on the highest query coverage and query TM-score, for each Zpa796 protein used as a query. Of the 670 AF2-predicted structures, 402 (60.5%) were assigned to known protein folds based on structural similarity to at least one of the experimentally-derived structural databases (PDB, CATH, SCOPe, or ECOD), while additional 77 predicted structures had hits in the AlphaFoldDB (AFDB, subsets “Proteome” and “Swiss-Prot”) (Fig. 2A, Supplementary Material 1: Table S4). Across retained best hits, mean query-normalized TM-scores ranged from 0.636 to 0.761, with a high mean query coverage (0.904 to 0.982) (Supplementary Material 2: Fig. S5), supporting a high extent of fold-level similarity between *Z. passerinii* Zpa796 secreted proteins and structural database entries. We note that proteins of intermediate length (101–600 amino acids) more often matched entries in the protein structural databases, consistent with their higher pLDDT confidence values in this size range (Fig. 2B-D, Supplementary Material 2: Figs. S6 and S7).

Among the secreted proteins, we predicted 310 effectors and 341 antimicrobial proteins (AMPs) (Supplementary Material 2: Fig. S4B-C, Supplementary Material 1: Table S1), using the machine learning pipelines EffectorP [19] and AMAPEC [38], respectively. Interestingly, a set of 141 proteins was predicted as both effectors and AMPs, that is, predicted to have both immune-suppressing and antimicrobial properties (Supplementary Material 2: Fig. S4D, Supplementary Material 1: Table S1); hereafter, we refer to these proteins as putative antimicrobial effectors. Within our putative effector and predicted AMP sets, we annotated 143 and 273 proteins, respectively, using protein structures, whereas we only annotated 75 effectors and 220 AMPs using a sequence-based approach (Supplementary Material 2: Fig. S8). Thus, structure-based data contributed to the functional annotation of effector-like proteins in *Z. passerinii*. A detailed description of the structure- and sequence-based annotations and their comparative overlap is provided as Supplementary Text in Supplementary Material 3. Per-protein annotations from both sequence- and structure-based methods are listed in Supplementary Material 1: Tables S1 and S4, respectively.

### Structural similarity network

**Fig. 3:**
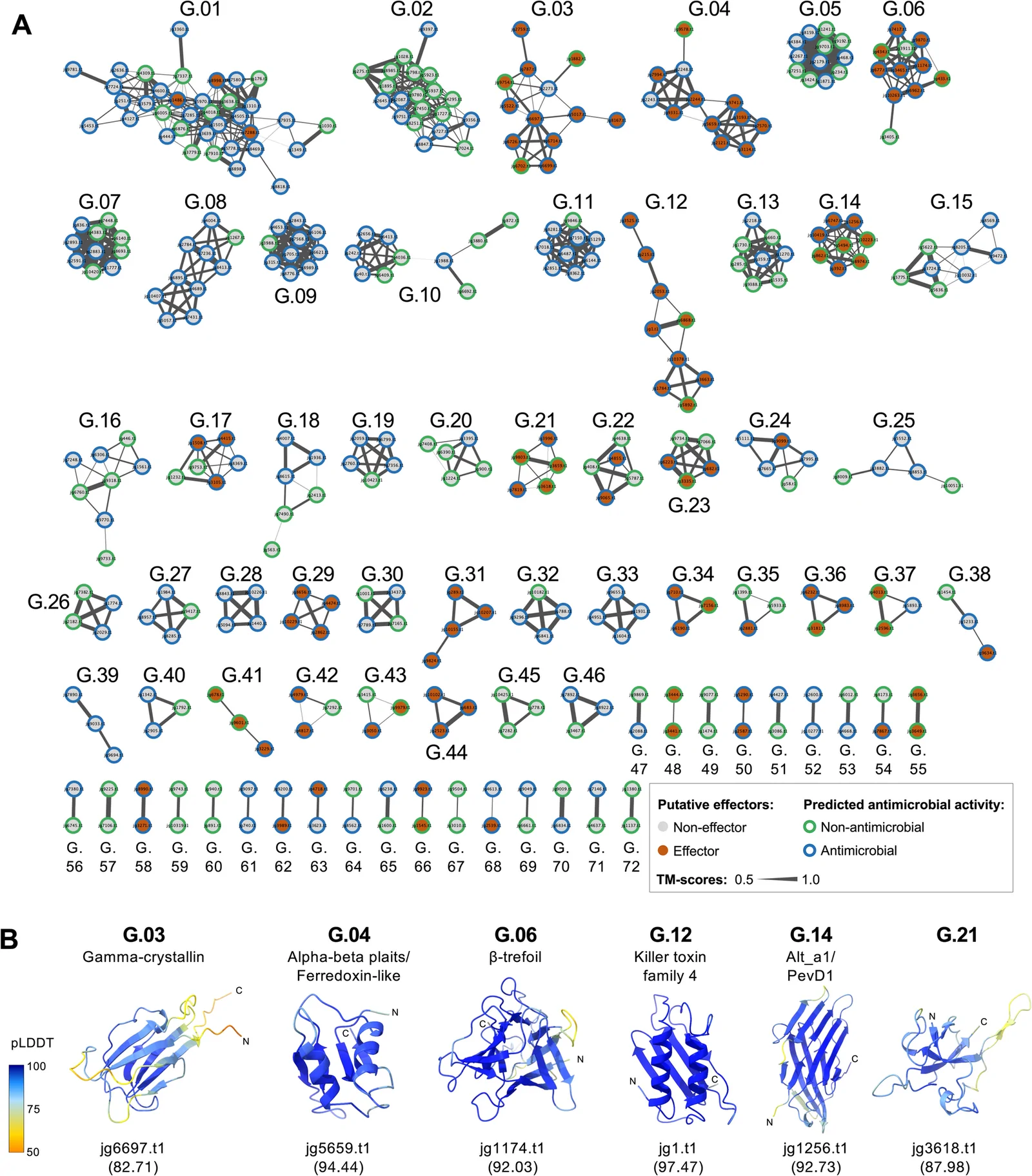
Overview of 72 structural clusters of the *Z. passerinii* Zpa796 secretome. (**A**) Graph view of the structural similarity network. The network consists of 72 subgraphs (namely G.01-G.72), each representing a structural cluster. Nodes represent individual proteins and are color-coded according to effector prediction and putative antimicrobial activity. Edges correspond to the structural similarity (TM-score) between pairs of proteins, with thicker edges indicating greater structural similarity. A bidirectional threshold of TM-score > 0.5 was applied to cluster two proteins as structurally similar. (**B**) Representative protein structures for each effector-enriched cluster (p-value_adj_ < 0.05). The representative structure corresponds to the node with the highest degree centrality. In cases where multiple proteins had the same centrality value, the representative was selected based on the structure with the highest mean pLDDT value (in parentheses). Letter codes: N, N-terminus; C, C-terminus

To further explore structural relationships among secreted proteins in *Z. passerinii* Zpa796, we modelled a structural similarity network using the AF2-predicted structures (Fig. 3A). We considered two proteins similar when bidirectional TM-scores > 0.5. The network consisted of 364 nodes, each representing a predicted secreted protein structure (Table 1), and 876 edges connecting these proteins according to their similarity. Importantly, 341 nodes (94%) corresponded to proteins with pLDDT > 70, indicating that the structural clusters were largely supported by confident structure models; the other 23 corresponded to peripheral, low-centrality nodes within their clusters.

**Table 1:** Overview of the proteins and clusters in the structural similarity network

Statistics	Count
Proteins	364
Structural clusters	72
Proteins in the largest structural cluster	36
Proteins in the smallest structural cluster	2
Putative effectors	107
Clusters containing putative effectors	31
Predicted AMPs	224
Clusters containing predicted AMPs	63

The network displayed a modular structure, with 72 connected components (subgraphs), each representing a structural cluster of proteins showing high structural similarity (Fig. 3A; Table 1, Supplementary Material 1: Table S4). The largest structural cluster comprised 36 proteins (nodes), while 26 structural clusters each contained only two proteins (Table 1). Of the 670 AF2-predicted structures used as input for the network analysis, 306 proteins that did not group into any structural cluster were considered as “singletons” (Supplementary Material 1: Table S4), that is, they lacked detectable structural similarity to other proteins in the Zpa796 secretome. Overall, the network topology reflects a modular secretome architecture in *Z. passerinii* Zpa796, with common folds forming structurally similar groups. In contrast, singletons may represent secreted proteins with unique structural features or proteins that do not adopt a rigid three-dimensional conformation under conditions captured by structure prediction methods. Detailed network statistics and robustness of the structural clusters are described in Supplementary Material 3.

### Functional landscape of structurally similar clusters and structural singletons

We investigated the putative functional context of each structural cluster by integrating our functional annotations (structure- and sequence-based) into the network.

Overall, structural clusters were consistent with fold-level annotations from the hierarchical structural databases we tested (CATH, ECOD, and SCOPe). When proteins in a given cluster were annotated using structures, they usually mapped to the same hierarchical category: ‘topology’ in CATH, ‘fold’ in SCOPe, and ‘X-group’ in ECOD (Supplementary Material 1: Table S4). Because these categories reflect similarities in secondary structure and folding patterns [39–42], this agreement indicates that each cluster represents a conserved structural group and may provide putative functional context for cluster members. Accordingly, clusters in the network spanned diverse protein families, including enzyme-associated clusters (e.g., peptidases, lipases, hydrolases, and oxidoreductases), transport- or binding-related proteins (e.g., immunoglobulin-like and LRR-like domains), fungal surface-associated families (e.g., hydrophobins), toxin-like proteins (e.g., killer toxin proteins), among others (Supplementary Material 1: Table S4). Nevertheless, five clusters (G.21, G.29, G.48, G.55, and G.58) were composed exclusively of uncharacterized proteins (Supplementary Material 1: Table S4). These clusters represent proteins that lack close structural homologs in current databases.

Putative effectors and predicted AMPs were distributed across the network (Fig. 3A). A total of 107 putative effectors and 224 predicted AMPs were distributed across 31 and 63 structural clusters, respectively (Table 1), indicating that diverse folds are potentially used to manipulate plant immunity and to interact with other microbiome members. Among these, 79 proteins were predicted as both effectors and AMPs, that is, putative antimicrobial effectors. Given this distribution, we next tested whether specific structural clusters were enriched, that is, had a higher-than-expected proportion of putative effectors or predicted AMPs (Supplementary Material 1: Table S5). We identified six structural clusters enriched with putative effectors (p-value_adj_ < 0.05) (Fig. 3B, Supplementary Material 1: Table S5): G.03 (Gamma-crystallin proteins), G.04 (Alpha-beta plaits/Ferredoxin-like), G.06 (β-trefoil), G.12 (Killer toxin family 4, KP4), G.14 (Allergen Alt a1, Alt_a1), and G.21 (uncharacterized proteins) (Fig. 3B, Supplementary Material 1: Table S4). No structural cluster was significantly enriched with AMPs (p-value_adj_ > 0.05) (Supplementary Material 1: Table S5) and predicted AMPs were broadly distributed across the different protein folds in the structural network (Fig. 3A). However, clusters with the highest proportion of proteins exclusively predicted as AMPs were usually associated with enzymatic activities (Odds ratios > 2 for AMPs; Supplementary Material 1: Table S5). These include G.08 (Peptidase S8), G.09 (Chloroperoxidases), G.11 (Acid proteases), and G.28 (Peptidase S28), among others (Supplementary Material 1: Table S4). Interestingly, three of the effector-enriched clusters (G.03, G.04, and G.12) also showed a high proportion of putative antimicrobial effectors despite no AMP enrichment (Odds ratios > 2 for AMPs, Supplementary Material 1: Table S5).

Among structural singletons, 218 (71%) lacked structural annotation in the three hierarchical structure databases (CATH, SCOPe, and ECOD) (Supplementary Material 1: Table S4). Moreover, many of these singletons contain more than 40% disordered residues or show lower mean pLDDT values defined by AF2 (Supplementary Material 2: Fig. S2), suggesting that many singletons in the secretome may represent poorly characterized folds or proteins with disordered regions. Among the remaining 88 singletons, some of the assigned folds included Rossmann-like, immunoglobulin-like, phosphorylase/hydrolase-like, and β-propeller folds (Supplementary Material 1: Table S4). A total of 62 putative antimicrobial effectors were included among the structural singletons.

In summary, we found that putative effectors and predicted AMPs from *Z. passerinii* Zpa796 are distributed across diverse protein folds. Effector-enriched clusters included proteins similar to toxin-like and interaction-associated proteins from databases, suggesting a role of these proteins in host immune interference; meanwhile, clusters with the highest proportions of AMPs were mainly linked to enzymatic activities. An overview of other structural clusters is provided in Supplementary Material 3.

### Physicochemical properties across proteins in the network

Since predicted AMPs were dispersed across the structural similarity network, we asked whether predicted antimicrobial activity of *Z. passerinii* proteins is better explained by physicochemical properties than by fold similarity. We evaluated whether a defined set of physicochemical properties distinguished putative antimicrobial effectors from proteins predicted only as effectors or AMPs. We compiled six properties: mature protein length, net charge (pH 7), charge density (net charge per residue), maximum helical hydrophobic moment, mean hydrophobicity, and surface hydrophobicity. We focused on these properties as they capture key biophysical features that contribute to the activity of small secreted proteins, including electrostatic interactions with charged surfaces, membrane association, and protein–protein interactions [43].

**Fig. 4:**
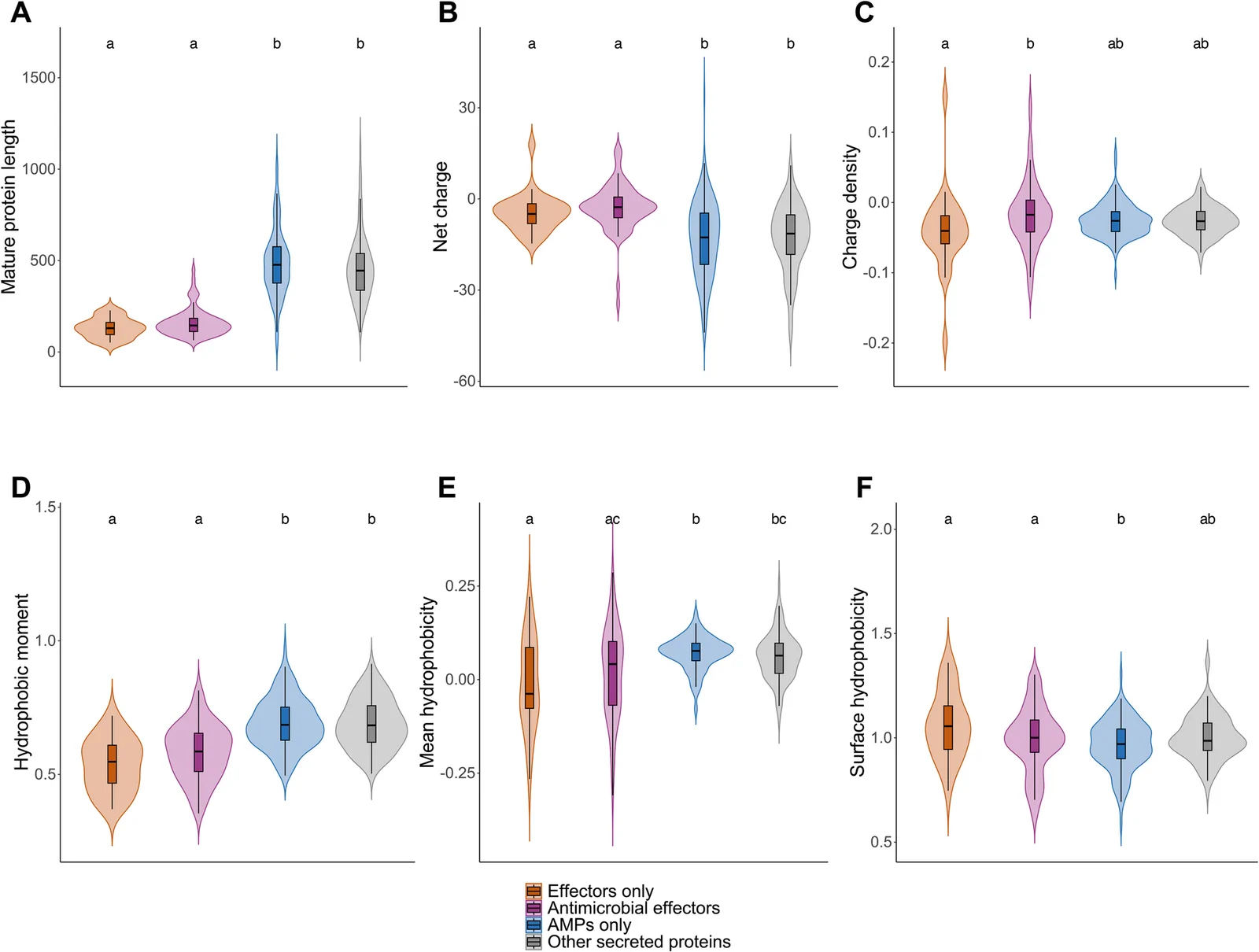
Comparison of physicochemical properties among functional protein groups within the structural similarity network of the *Z. passerinii* Zpa796 secretome. (**A**) Mature protein length (number of amino acid residues), (**B**) Absolute net charge (pH 7), (**C**) Charge density (net charge normalized by the number of residues), (**D**) Hydrophobic moment (maximum local helical amphipathicity), (**E**) Mean hydrophobicity, and (**F**) Surface hydrophobicity. For each property, groups sharing the same letter indicate no significant difference according to Dunn’s test (p-value_adj_ > 0.05). Numbers of proteins: 28 putative effectors only, 79 putative antimicrobial effectors, 145 predicted only as AMPs, and 112 other secreted proteins

To assess if predicted antimicrobial proteins and putative effectors would differ according to their physicochemical properties, we first grouped proteins from the network into four categories: putative antimicrobial effectors (*N* = 79), proteins predicted solely as putative effectors (*N* = 28), proteins predicted only as AMPs (*N* = 145), and other secreted proteins (*N* = 112). The four functional groups exhibited significant differences (p-value < 0.05, Kruskal-Wallis test) across all six properties (Fig. 4, Supplementary Material 1: Table S6). Therefore, we tested pairwise differences among groups using Dunn’s test. The putative effector-like proteins (predicted as antimicrobial effectors or effectors only) in the network were the shortest and had higher absolute net charge than proteins predicted only as AMPs and other secreted proteins (p-value_adj_ < 0.05) (Fig. 4A-B, Supplementary Material 1: Tables S7 and S8), consistent with small size and higher electrostatic attraction of the effector-like proteins. However, the net charge values inflated with increasing protein length (Supplementary Material 2: Fig. S10); thus, we also calculated the charge density (net charge per number of residues) to correct for length differences and enable a more comparable measure of the charge across proteins. Following length normalization, putative antimicrobial effectors exhibited higher charge density than putative effectors (p-value_adj_ < 0.05), although charge densities overlapped across the other groups (Fig. 4C, Supplementary Material 1: Tables S7 and S8). Interestingly, this pattern is consistent with a shift in amino acid composition toward cationic residues in putative antimicrobial effectors relative to proteins predicted solely as effectors. We also compared different measures of hydrophobicity. The surface hydrophobicity was lower in proteins predicted solely as AMPs, whereas hydrophobic moment and mean hydrophobicity were higher (p-value_adj_ < 0.05) (Fig. 4D–F, Supplementary Material 1: Tables S7 and S8), in agreement with a greater membrane affinity via amphipathic segregation in predicted AMPs than in the set of putative effector-like proteins (antimicrobial effectors and effectors only).

We repeated the comparisons for each physicochemical property among structurally similar clusters, limiting the analysis to the 46 clusters (63.9% of the 72 total) with at least three protein members (Supplementary Material 2: Fig. S11, Supplementary Material 1: Tables S9-S11). We found significant differences for all six properties in these analyses (p-value < 0.05, Kruskal-Wallis test) (Supplementary Material 1: Table S9). The physicochemical properties that distinguished more pairs of structural clusters were mature protein length, followed by net charge, hydrophobic moment, and mean hydrophobicity (Supplementary Material 2: Fig. S11, Supplementary Material 1: Table S11). Furthermore, several clusters also showed wide within-cluster spreads across the six properties (Supplementary Material 2: Fig. S11), reflecting variation in the physicochemical properties among proteins within the same fold group. Overall, we found significant pairwise differences (p-value_adj_ < 0.05, Dunn’s test) between clusters predominantly composed of putative effector-like proteins and clusters composed of predicted AMPs or other secreted proteins (Supplementary Material 1: Table S11). We reported the results of physicochemical properties among structural clusters of *Z. passerinii* secretome in Supplementary Material 3.

### Conserved structural scaffolds support sequence diversification

**Fig. 5:**
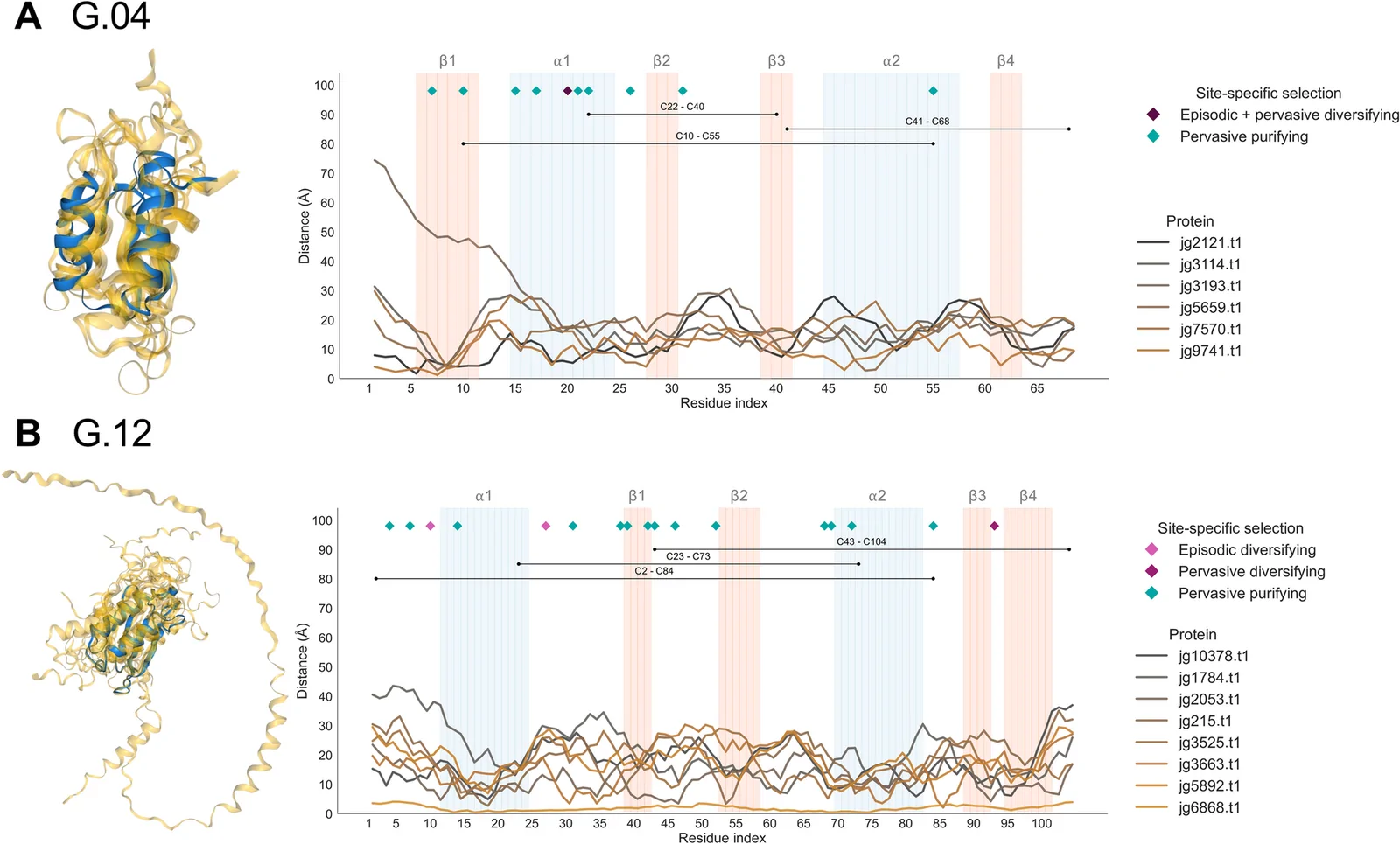
Structural alignments of proteins within structural clusters (**A**) G.04 and (**B**) G.12. On the left, multiple structural alignments with the representative core protein highlighted in blue (jg2244.t1 for G.04, jg1.t1 for G.12, with each representative protein corresponding to the shortest protein within the group). On the right, charts showing the distances (in angstroms) between Cα atoms of aligned residues for each cluster member compared to the core protein. Pairs of cysteines forming disulfide bonds and segments forming secondary structures (α-helices in blue and β-strands in red) are highlighted for the core protein of each cluster. Sites under selection are indicated by diamonds, with magenta shades representing diversifying selection and cyan denoting sites under purifying selection

Despite the structural similarity of proteins within clusters, their physicochemical properties varied; we therefore hypothesized that small, localized structural differences among proteins within the same cluster may contribute to protein diversification. To explore this, we focused on effector-enriched clusters G.04 and G.12 (Fig. 5, Supplementary Material 2: Fig. S12-S14), both containing putative antimicrobial effectors and showing within-cluster variation in physicochemical properties.

The G.04 cluster comprises proteins annotated as alpha-beta plaits (CATH fold and ECOD X-group) or ferredoxin-like (SCOPe fold), while the G.12 cluster comprises proteins annotated as KP4-like proteins (Fig. 3B, Supplementary Material 1: Table S4). For G.04, protein members varied in structural similarity, so we focused on seven representative proteins that formed the most tightly connected subgroup and were more structurally similar (Fig. 5A, Supplementary Material 2: Fig. S12). Both G.04 and G.12 clusters consisted of proteins with an α + β organization, meaning they typically contained α-helices and β-strands alternating in a stable scaffold (Fig. 5). The core structure of G.04 aligned proteins (represented by protein jg2244.t1) was organized in a β-α-β-β-α-β arrangement, with five loop regions (3–9 residues) connecting the secondary structure elements (Fig. 5A). In the representative G.04 protein, we predicted six cysteines forming three disulfide bridges: C10–C55 (5.23 Å between α-carbons, Cα), C22–C40 (5.93 Å), and C41–C68 (6.42 Å) (Fig. 5A), which contribute to fold stability. Within G.04, the first (β1) and third (β3) β-strands exhibited the smallest distance variation between aligned amino acids, indicating the most conserved secondary elements among proteins. Meanwhile, the loops β1–α1 and β2–β3 displayed the largest deviations from the core protein fold (jg2244.t1, Fig. 5A). Among G.12 aligned proteins, the core structure (represented by jg1.t1) displayed an α-β-β-α-β-β organization, connected by five loops (2–14 residues) (Fig. 5B). In the representative G.12 protein, we also predicted six cysteines forming three disulfide bridges: C2–C84 (5.10 Å between Cα), C23–C73 (5.72 Å), and C43–C104 (6.40 Å) (Fig. 5B). Among G.12 proteins, the smallest distances between aligned residues were observed within the α-helices (α1 and α2) and the largest in the loop α1–β1, when compared to the core protein fold (jg1.t1, Fig. 5B).

Despite structural similarity, we found low sequence conservation within G.04 and G.12 clusters (Supplementary Material 2: Figs. S12 and S13). In G.04, four cysteines were conserved across all members, whereas most other positions varied and included conservative and non-conservative replacements (Supplementary Material 2: Fig. S12A). Meanwhile, in G.12, sequence conservation was even lower, with only one cysteine and one tryptophan conserved across all sequences (Supplementary Material 2: Fig. S12B). Overall, the sequence alignment patterns mirrored the topology within structural clusters in the network, yet structural similarity exceeded sequence identity between pairs of proteins (Supplementary Material 2: Fig. S13). Even pairs of proteins with nearly identical structures displayed several non-conservative amino acid replacements, resulting in low sequence identity, for example paralogs jg1.t1 (putative antimicrobial effector) and jg6868.t1 (putative effector only) (RMSD = 0.46 and TM-score = 0.986, normalized for both proteins) from the G.12 cluster (Supplementary Material 2: Figs. S12B and S13B).

To further evaluate whether sequence variation within these structurally conserved clusters showed signatures of constraint or diversification, we performed a codon-level selection analysis for the Zpa796 proteins in G.04 and G.12. Most sites predicted to be under selection were consistent with a purifying selection scenario and included residues located within secondary-structure elements and disulfide-bonded cysteines important for fold stabilization (Fig. 5, Supplementary Material 2: Fig. S14). Conversely, sites predicted to be under diversifying selection mapped to surface-exposed regions, including one site in the α1 helix of G.04 and three sites located in loops of G.12 (Fig. 5, Supplementary Material 2: Fig. S14). Together, these results indicate that G.04 and G.12 proteins retain constrained, disulfide-stabilized structural cores, with low sequence conservation. Thus, variation arose from amino acid replacements that contribute to differences in surface properties, consistent with these stable cores serving as scaffolds that allow sequence diversification that may contribute to functional divergence.

### Protein evolution among homologs of *Z. passerinii *and other *Zymoseptoria *spp.

To gain insights into the evolution of the structurally similar proteins across species, we searched for homologs within the *Zymoseptoria* genus. We used OrthoFinder to identify homologous relationships between secreted proteins of *Z. passerinii* Zpa796 and other *Zymoseptoria* species. Of the 364 proteins in the structural similarity network, 359 had homologs in at least one other species (Supplementary Material 1: Table S12). Overall, most proteins from Zpa796 within a given structural cluster fell into different orthogroups along with homologs from the other species (Supplementary Material 1: Tables S4 and S12), indicating that their primary sequences have diverged greatly both within *Z. passerinii* and across closely related species.

**Fig. 6:**
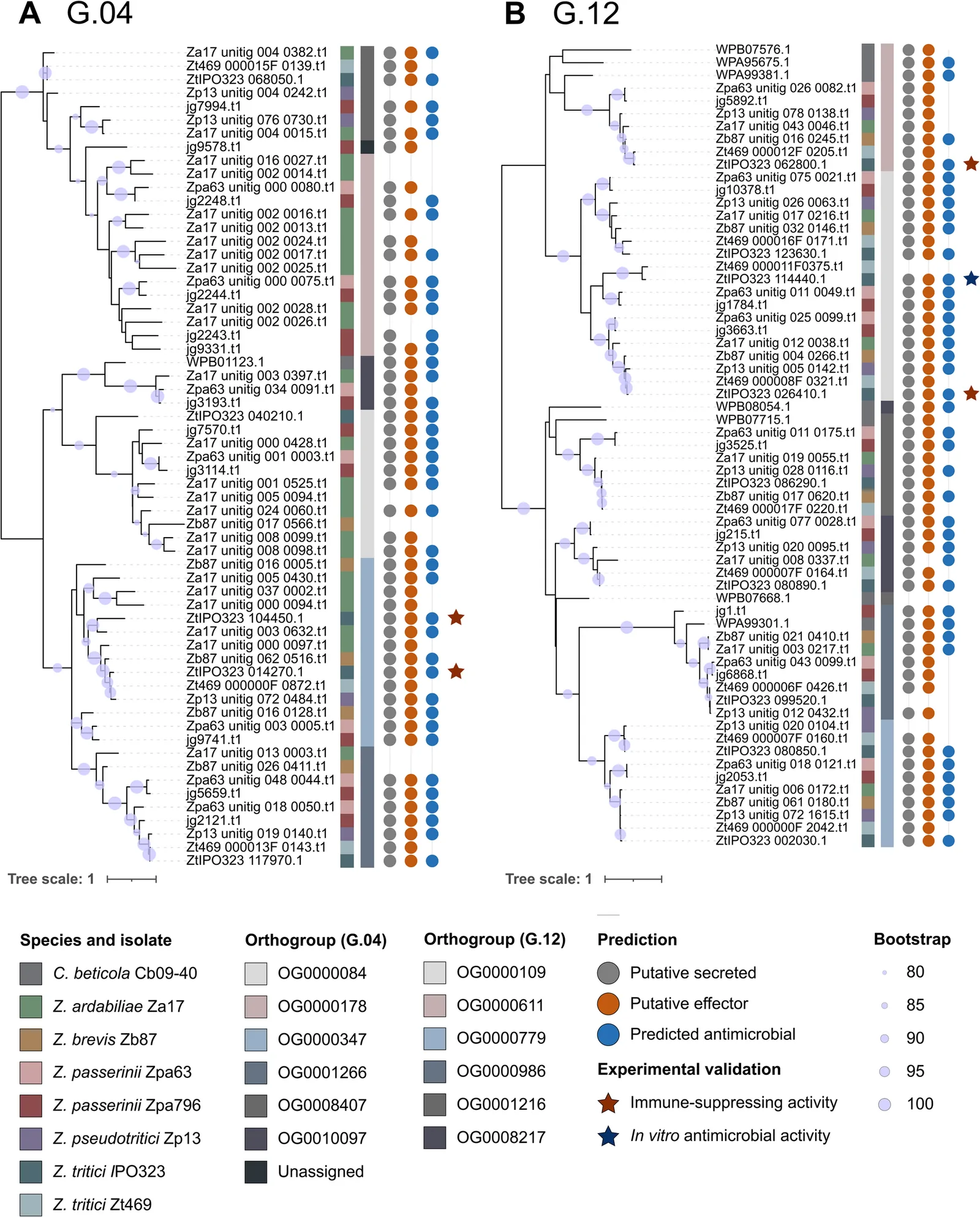
Phylogenetic relationships among proteins from *Z. passerinii* Zpa796 and their homologs across *Zymoseptoria* species. Each phylogeny includes proteins from the structurally similar clusters (**A**) G.04 and (**B**) G.12. Unrooted maximum-likelihood phylogenies (consensus trees), with *Cercospora beticola* Cb09-40 used as the outgroup. Stars indicate effectors from *Z. tritici* IPO323 reported to suppress plant immune responses [60] or to display in vitro antimicrobial activity [61]. Proteins sharing the same orthogroup indicate some sequence conservation among homologs. Branch lengths are drawn to scale; nodal support values are given as local bootstrap values above the branches (when > 80). The scale bar corresponds to the expected number of substitutions per site

**Fig. 7:**
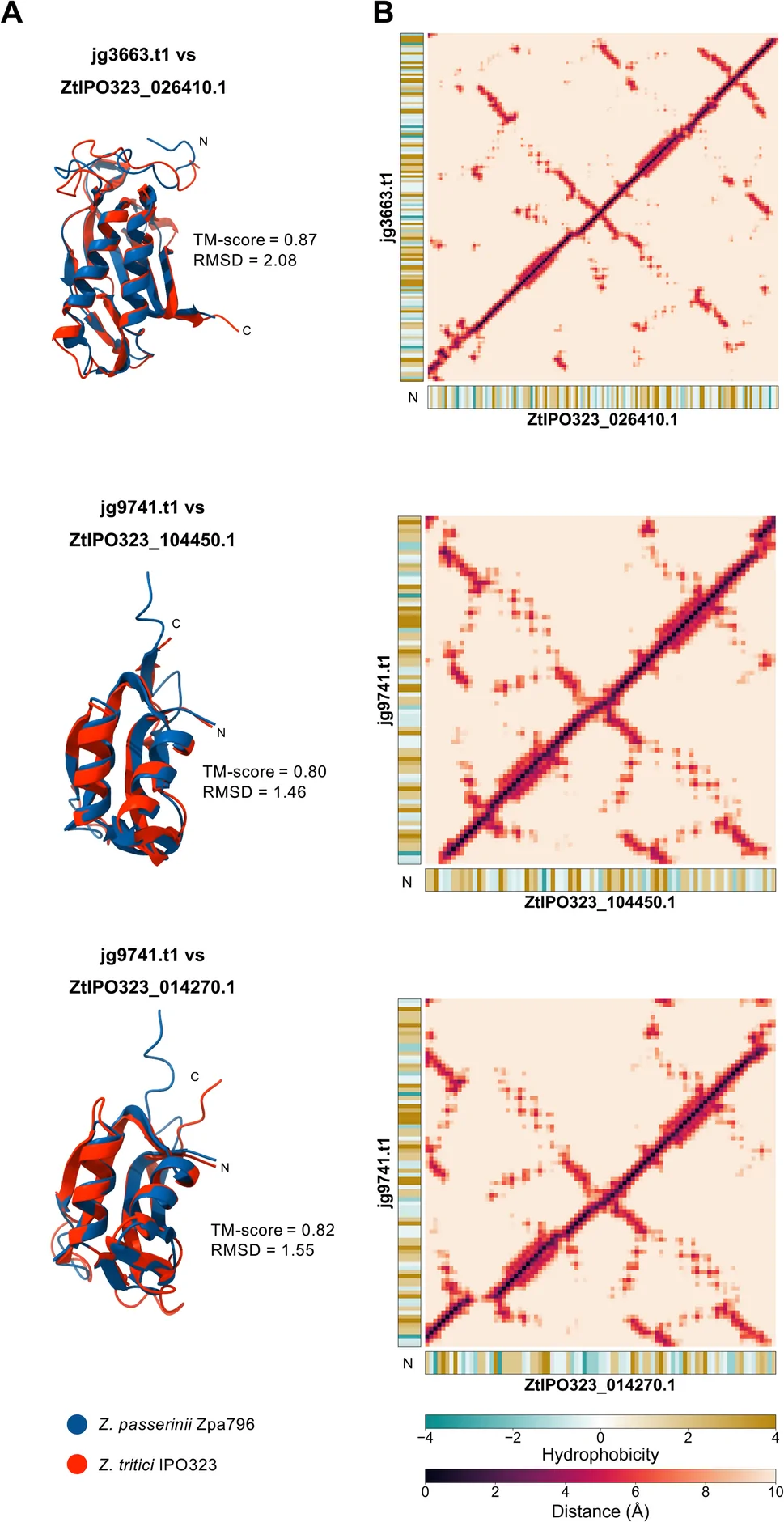
Structural comparison between*Z. passerinii*Zpa796 proteins and their homologs in*Z. tritici*IPO323**.** The alpha-beta plait (jg9741.t1) and KP4-like (jg3663.t1) proteins from *Z. passerinii* Zpa796 belong to effector-enriched structural clusters G.04 and G.12, respectively. (**A**) Superposition of tertiary structures, with TM-scores normalized to the *Z. passerinii* Zpa796 proteins (shown in blue). (**B**) Symmetric distance matrix comparing all pairs of amino acid residues, measured using Cα atoms as reference. Helices are represented as strips directly adjacent to the diagonal, while antiparallel β-strands appear as cross-diagonal patterns. Hydrophobicity per residue was calculated using the Kyte-Doolittle scale. Proteins from *Z. passerinii* Zpa796 are displayed on the y-axis, and proteins from *Z. tritici* IPO323 (according to [60]) on the x-axis. The letter N indicates N-termini

We selected the effector-enriched clusters G.04 and G.12 to investigate the evolutionary relationships between Zpa796 proteins and their homologs in other *Zymoseptoria* species (Fig. 6). Homologs from both clusters were broadly distributed across the genus, with different levels of gene duplication among species (Fig. 6). This suggests that the proteins represented by G.04 and G.12 are evolutionarily conserved across *Zymoseptoria*, with lineage-specific expansion of these families in some species. Most homologs were also predicted to be secreted antimicrobial effectors, supporting the conservation of both structural and putative functional features across species. We next used proteins jg9741.t1 and jg3663.t1 (clusters G.04 and G.12, respectively) for detailed structural comparison with their homologs in the reference isolate *Z. tritici* IPO323 (Fig. 7). Protein jg3663.t1 had a close homolog in IPO323 (ZtIPO323_026410.1), while jg9741.t1 had two paralogs: ZtIPO323_014270.1 and ZtIPO323_104450.1 (Fig. 6, Supplementary Material 1: Table S12). See Supplementary Material 1: Table S13 for aliases of these IPO323 proteins across annotation versions. Pairwise structural alignments of homologs revealed highly similar folds, with small differences found in surface-exposed loops and terminal extensions (Fig. 7), supporting overall fold conservation of these proteins across the sister species. Additional information on the distribution of homolog and alignment scores is provided in Supplementary Material 3.

We next assessed whether sequence divergence between species leads to differences in protein properties. We compared physicochemical properties associated with antimicrobial activity for jg3663.t1 and jg9741.t1 and their homologs in *Z. tritici* IPO323 (Supplementary Material 1: Table S14). Both Zpa796 proteins and their IPO323 homologs were annotated as antimicrobial effectors. The main differences among homologs were in net charge and isoelectric point (pI), with the *Z. passerinii* Zpa796 proteins exhibiting higher net charges and pI values than their counterparts in *Z. tritici* IPO323 (Supplementary Material 1: Table S14). Accordingly, the homologs from Zpa796 should remain more positively charged in the mildly acidic host apoplast than the homologs from IPO323. Although hydrophobic moment was comparable across homologs of the two species, jg9741.t1 (G.04) and ZtIPO323_014270.1 displayed a more hydrophobic nonpolar face for helix α1 (amphipathic helix) (Supplementary Material 2: Fig. S15) and higher surface hydrophobicity than the IPO323 paralog ZtIPO323_104450.1 (Supplementary Material 1: Table S14). Mean hydrophobicity and Boman index were broadly similar across homologs (Supplementary Material 1: Table S14). Together, these data indicate species- and paralog-specific variation in surface properties for these proteins with conserved structural scaffolds.

## Discussion

We explored the structural landscape of secreted proteins in the wild grass pathogen *Z. passerinii* using an array of protein structure analyses and described common folds in sequence-divergent proteins that may engage in host manipulation and plant-associated microbial interactions. An interesting finding is that a large proportion (> 45%) of all secreted proteins are predicted to encode antimicrobial proteins, pointing to an unacknowledged importance of microbial interactions during the life cycle of *Z. passerinii*. Not all predicted AMPs are necessarily produced to antagonize other microbes, but this suggests a prevalence of features associated with antimicrobial potential among the secreted proteins. Furthermore, many putative effectors are also predicted to be antimicrobial, defining a valuable set of candidates for future functional assays.

To explore the secretome of *Z. passerinii*, we started our investigations with a detailed assessment and comparison of predicted protein structures. We first evaluated the structural predictions generated by AF2 and ESMFold and obtained models with higher confidence values for AF2 predictions, particularly for proteins with conserved domains. In contrast, predictions for short proteins were more variable, with AF2 leaving some sequences unmodeled and ESMFold assigning very low-confidence models. These unmodeled sequences were frequently predicted as intrinsically disordered, consistent with more dynamic conformational behavior [44] that could, for example, contribute to molecular mimicry or membrane translocation of small secreted proteins during host infection [45, 46].

Protein annotation and network-based clustering of AF2-predicted structures revealed the relationships across proteins of the secretome of *Z. passerinii* Zpa796. The fragmented topology of the structural similarity network reflected a broad structural diversity among the secreted proteins. Instead of forming a densely connected architecture, structurally similar proteins grouped into discrete clusters corresponding to protein families. Among the effector-enriched clusters were two families, Alt a1–like (G.14) and β-trefoil (G.06), containing folds previously implicated in host immune modulation by fungal plant pathogens [47, 48]. Alt a1–like proteins can interfere with ROS signaling [49], inhibit antifungal pathogenesis-related proteins [50], and show induced gene expression in *Zymoseptoria* species during host colonization [51]. Meanwhile, β-trefoil proteins are commonly associated with fungal lectins and non-enzymatic protease inhibitors involved in diverse ligand interactions [52–54].

Other effector-enriched folds in the secretome of *Z. passerinii* Zpa796 correspond to proteins with the potential to engage both in host defenses and microbial interactions. These folds encompassed KP4-like (G.12) [55–57], alpha-beta plait (ferredoxin-like; G.04) [58, 59], and gamma-crystallin-like (G.03) [59] proteins. Crystallin-like effectors are widespread across fungi [6, 20, 21], and usually display induced gene expression during early host infection [59]. Meanwhile, KP4-like effectors contribute to ion channel interference, as shown in *U. maydis* [57], and PAMP-triggered immunity (PTI) suppression, as shown in *Z. tritici* [60] and *F. graminearum* [55]. Similarly, alpha-beta plaits, which include some variants such as the killer toxin family 6 (KP6)-like subfamily, exhibit functional divergence across fungi, being able to modulate effector-triggered immunity [60] or inducing host cell death [59]. Moreover, both KP4-like and KP6-like variants of alpha-beta plaits from the sister species *Z. tritici* may have antifungal activity in vitro [61]. Therefore, the plethora of putative antimicrobial effectors within these effector-enriched clusters from *Z. passerinii* suggests that these fungal proteins may have evolved multifunctionality in plant-associated niches [15, 62, 63].

Interestingly, some effector-associated clusters remained structurally unclassified despite structural similarity among members (i.e., G.21, effector-enriched). In these cases, proteins showed a prevalence of intrinsically disordered regions that contribute to structural flexibility and interactions with multiple molecular targets, which can lead to neo-functionalization of some proteins [64]. It is plausible to consider that the abundance of disordered proteins in the secretome of *Z. passerinii* Zpa796 also contributes to the evolutionary diversification of proteins involved in host–microbe interactions and this may lead to functional versatility by these proteins.

Unlike putative effectors, predicted AMPs did not achieve fold-level enrichment, suggesting that antimicrobial activity depends on physicochemical properties recurring across diverse protein folds. A key discriminator between proteins predicted exclusively as AMPs and putative effector-like proteins was helical amphipathic patterning, measured by the hydrophobic moment. Amphipathic α-helices are associated with antimicrobial activity due to their ability to interact with and damage microbial membranes [43, 65–67]. In *Z. passerinii* Zpa796, greater hydrophobicity and enhanced amphipathicity among proteins predicted exclusively as AMPs indicate amphipathic membrane interactions [67] by these proteins. Conversely, effector-like proteins were shorter and showed higher absolute net charge compared to other secreted proteins. Notably, putative antimicrobial effectors exhibited greater charge density (net charge per residue), reflecting an enrichment in positively charged residues compared with proteins predicted exclusively as effectors. Together, these shifts in hydrophobic moment and charge density suggest different interaction modes, with AMP-only proteins more consistent with membrane insertion and disruption, and antimicrobial effectors potentially having stronger electrostatic attraction to negatively charged targets, including microbial membrane lipids [43, 67] and host cell-wall components [68, 69]. These molecular interactions remain to be tested experimentally.

The physicochemical differences, together with the abundance of putative antimicrobial effectors across the structural similarity network, suggest that antimicrobial properties repeatedly emerged within effector-enriched folds. This interpretation is further supported by the phylogenetic and selection analyses, showing that G.04 and G.12 homologs are broadly distributed across *Zymoseptoria* species, while residues under diversifying selection are mainly associated with more variable surface-exposed regions and residues under purifying selection are consistent with maintenance of a constrained structural core [21, 63]. Thus, two complementary mechanisms may contribute to the functional diversification of effector-like proteins in *Z. passerinii* while retaining common protein folds: (i) non-conservative amino acid replacements and (ii) the maintenance of flexible loop and linker regions.

First, our findings are consistent with putative antimicrobial effectors emerging through amino acid replacements on effector-enriched structural scaffolds. As a result, antimicrobial properties in effector-like proteins may have arisen through amino acid replacements that alter the surface electrostatic potential, while effector backbones remain constrained. Likewise, amino acid substitutions that reconfigure surface properties are recurrent in common effector folds across fungal plant pathogens [21, 69–71]. Nevertheless, alternative evolutionary routes are plausible, including antimicrobial effectors evolving from ancestral AMPs that later diversified toward host manipulation [15]. Thus, while our dataset supports effector-scaffold diversification as the dominant pattern in *Z. passerinii* Zpa796, it does not exclude AMP-derived trajectories in specific proteins.

Second, disulfide-stabilized constrained structural cores paired with flexible loops and linker regions contribute to diversification within common effector families. Although G.04 (alpha–beta plait/ferredoxin-like) and G.12 (KP4-like) proteins retained highly similar core folds, we observed most structural variation in loop regions. Variation in loop length and amino acid composition can influence ligand binding and molecular recognition [72–74]. Similarly, loop flexibility has been linked to functional shifts in fungal secreted proteins, including cerato-platanins from *Ustilago* spp [75] and the acquisition of antimicrobial properties by a group of fungal chitinases [76]. In *Z. passerinii* Zpa796, the combination of a stable structural core and flexible loop regions likely enables these proteins to maintain their overall fold while contributing to new putative functions or allowing different binding partners throughout host infection.

Comparing putative antimicrobial effectors of *Z. passerinii* Zpa796 with effector homologs in *Z. tritici* IPO323 shed further light on how structurally similar effectors evolve and diversify across related fungal pathogens. We focused on the Zpa796 proteins jg3663.t1 (a KP4-like protein, G.12) and jg9741.t1 (an alpha–beta plait protein, G.04) for their placement in effector-enriched clusters and predicted antimicrobial activity. Notably, their homologs in IPO323 are effectors that induce plant-immune suppression [60], providing a functional framework for our findings in Zpa796. Despite high structural similarity, differences in surface-exposed regions and amino acid composition among *Z. passerinii* Zpa796 and *Z. tritici* IPO323 homologs may also reflect host adaptation. Although the leaf apoplast of grasses is mildly acidic during biotic stress [77, 78], higher isoelectric point (pI) and net charge of Zpa796 proteins relative to their IPO323 counterparts likely reflect host-specific tuning of the surface charge, enhancing interactions with anionic cell-wall components and microbial membranes in the barley apoplast when compared with conditions experienced by *Z. tritici* in wheat. Experimental assays in the native host or with plant-specific receptors are necessary to test this hypothesis.

Notably, amphipathic α-helices were an additional characteristic of jg9741.t1 from Z. *passerinii* Zpa796 and its homologs in *Z. tritici* IPO323, all adopting alpha–beta plait folds. IPO323 homologs are KP6 variants within the alpha-beta plaits group [60], in which amphipathic α1 helices contribute to the disruption of microbial membranes [79]. Interestingly, we also identified a recurrent signal of diversifying selection in the α1 helix of these proteins in Zpa796, supporting this region as a hotspot of adaptive evolution and suggesting that these changes may modulate surface properties linked to membrane interaction. Preliminary confrontation assays showed that homolog ZtIPO323_014270.1 inhibits in vitro growth of *Escherichia coli* (Thynne et al., unpublished), further supported by the antimicrobial activity among killer toxin–like effectors from *Z. tritici* [61]. Together, these experimental findings support our in silico analyses of putative antimicrobial effectors in *Z. passerinii* and validate our list of candidates for future experiments aimed at elucidating how these proteins interact with microbial membranes or host components.

We here present a computational prediction and analysis of the secretome of *Z. passerinii*. Our findings provide novel insights into the evolution of secreted proteins within and between species. Furthermore, the catalogue of protein structures described here represents a foundation to prioritize candidate effector families for future validation in the species. We note that the assignment of effector-like and antimicrobial properties may include false positives. Therefore, future studies focusing on the characterization of the candidates we describe here could include antimicrobial assays against competitor microbes and plant immune-suppression assays, as described for the sister species *Z. tritici* [60, 61]. Additionally, targeted mutational analyses and molecular dynamics simulations could help to elucidate the contribution of common protein folds and selected residues to protein function.

## Conclusions

Using a suite of deep learning tools, we find that the secretome of the fungal pathogen *Z. passerinii* comprises a diverse repertoire of proteins that appear to have evolved from common protein folds. Within groups of structurally similar proteins, local variation in surface-exposed regions and physicochemical properties likely provides a basis for protein evolution that can lead to functional diversification, including host-targeting activities and antimicrobial properties. Our findings suggest that a large proportion of proteins secreted by fungal pathogens may contribute to interactions with other microorganisms occupying the same niche. Together, this secretome-wide study provides a valuable source of effector candidates for experimental functional elucidation and a general strategy that can be transferred to the study of secretomes from other eukaryotic microorganisms.

## Methods

### Assembly of genomic and crystallized protein structure data

We retrieved the genome sequence, gene models, and 10,461 protein sequences of *Z. passerinii* isolate Zpa796 from [34] (Supplementary Material 1: Table S1). Zpa796 was obtained from wild barley (*Hordeum murinum* subsp. *glaucum*) sampled in Iran, as reported in [34]. From the Protein Data Bank (PDB v240622 [37]), we retrieved a dataset of 228 fungal proteins, consisting of crystallized structures and amino acid sequences (Supplementary Material 1: Table S3). We filtered entries in the PDB using the text terms “fungal effector” and “fungal protein.” Only proteins from fungal organisms within the phyla Ascomycota and Basidiomycota were kept. Structures crystallized with a single chain were retained, and the final dataset was manually curated to reduce protein redundancy.

### Sequence-based protein functional annotation

We performed sequence-based annotation of the *Z. passerinii* Zpa796 proteome to identify conserved protein domains and functional signatures. Protein domains were predicted using InterProScan v5.54-87.0 [80]. EuKaryotic Orthologous Groups (KOG), Gene Ontology, and KEGG annotations were retrieved using eggNOG-mapper v2 [81]. Functional classes such as CAZymes, lipases, and proteases were identified based on hidden Markov model (HMM) or BLASTp searches against specialized databases. Secondary metabolite biosynthesis clusters were predicted using antiSMASH v7.1.0 [82]. Detailed parameters, database versions, and thresholds are provided in Supplementary Material 3.

### Secretome and effector prediction

We defined the predicted secretome by the presence of a signal peptide, predicted by SignalP v6.0 [83] and Phobius v1.01 [84], and the absence of transmembrane domains, predicted by TMHMM v2.0 [85] and Phobius. We used TargetP v2.0 [86] and DeepLoc v2.0 [87] to predict the subcellular localization of the secreted proteins. ESpritz v1.3 [88] was used to search for intrinsically disordered regions in the candidate secreted proteins. Fungal effectors are typically soluble secreted proteins that enter the apoplast or are delivered inside host cells [4, 19]. We defined the putative effectors of *Z. passerinii* Zpa796 from the predicted secretome using EffectorP v3.0 [19] set as fungi. EffectorP classifies secreted proteins as putative effectors using a probability threshold of > 0.5, with a reported accuracy of 90% [19].

### Structure prediction

Protein structures for the candidate secreted proteins were obtained using the mature sequences, with predicted signal peptides removed prior to modeling. We conducted structural prediction locally using AlphaFold2 (AF2) v2.3.1 [24] with the parameter “--max_template_date=2020-05-01” and ESMFold v1.0.3 [25] with “--chunk-size 32.” For AF2, five models were generated for each protein, and we selected the highest-quality model (ranked_0.pdb) based on the highest average pLDDT (predicted local distance difference test) score.

### Comparison of AlphaFold2 and ESMFold predictions

Fungal secreted proteins are highly variable in size, and many fungal proteins involved in plant–microbe interactions correspond to short amino acid sequences [4, 17, 19]. We compared structural predictions across the full secretome of *Z. passerinii* Zpa796 and also assessed prediction quality across protein length categories (in 100-residue intervals). For proteins predicted by both methods, we compared structural confidence (pLDDT values) and structural similarity. We used TM-align v20220412 [89] to calculate the TM-score and RMSD values. Additionally, we evaluated how similar the outputs from each method were to experimentally determined fungal structures by aligning predicted models to crystallized proteins retrieved from the PDB. Statistical analysis and comparison metrics are described in Supplementary Material 3.

### Structural annotations and antimicrobial activity prediction

We used the *easy-search* workflow from Foldseek [90] to perform structural similarity searches against the following protein structure databases: CATH50 v4.3.0 [39], SCOPe40 v2.08 [40], ECOD40 v291 [41], PDB v240101 [37] and AlphaFoldDB v4 (AFDB) [91] with separate runs for “Proteome” and “Swiss-Prot” entries. The Proteome section of the AFDB contained predicted structures for all proteins in reference proteomes, whereas the Swiss-Prot section included predicted structures from UniProt entries that were manually curated; we refer to these as AFDB (Proteome) and AFDB (Swiss-Prot), respectively. Searches were conducted with the parameters “-s 7.5 --alignment-type 1 --tmscore-threshold 0.5 --format-mode 4” and a customized “--format-output” parameter with several output columns, including “query, target, qcov, qtmscore”. We then parsed the best hit for each *Z. passerinii* Zpa796 protein based on the highest query coverage (qcov) and the highest query TM-score (qtmscore). Custom Python3 scripts were used to extract structural annotations from the best-hit entries in the reference databases. We predicted antimicrobial activity among candidate secreted proteins using the machine-learning pipeline AMAPEC v1.0b [38], with protein structure files generated by AF2 as inputs. AMAPEC calculates 70 sequence- and structure-derived variables and classifies antimicrobial activity with 89% accuracy for fungal proteins [38]. By default, AMAPEC uses a probability score > 0.5 to define a protein as antimicrobial, based on the rationale that protein structures with higher pLDDT values are more likely to reflect in vivo antimicrobial potential.

### Modelling and analysis of the structural similarity network

We built a structural similarity network to investigate protein homology relationships within the predicted secretome of *Z. passerinii* Zpa796. Structural similarity between secreted proteins was calculated using all-vs-all pairwise alignments with TM-align. Two structures were considered similar using as cutoff a TM-score > 0.5 normalized for both proteins, as values above this threshold are generally recommended for fold-level similarity between pairs of proteins [92]. We modeled the network using NetworkX [93] in Python3, based on the following criteria: (i) candidate secreted proteins were represented as nodes in the graph, with each node corresponding to one protein; (ii) connections between nodes were represented by edges when the bidirectional TM-score cutoff was met; the highest TM-score from each pairwise comparison was used as edge weight for plotting. We further analyzed the modeled network by calculating network statistics and metrics using NetworkX. The network was visualized using Cytoscape v3.10.3 [94]. We treated each network subgraph as an individual structural cluster, representing a group of proteins with similar structural features that might reflect functional or evolutionary relationships. To identify significant protein folds involved in *Z. passerinii* Zpa796 interactions with the host and other microorganisms during plant infection, we tested for enrichment of putative effectors and candidate antimicrobial proteins within structural clusters using Fisher’s exact test (p-value_adj_ < 0.05, using the Benjamini–Hochberg correction) from the SciPy v1.13.0 [95] library in Python3. To assess the robustness of the structural similarity network, we repeated the network analysis using subsets of AF2 models filtered by minimum mean pLDDT thresholds from 50 to 95 in steps of 5. For each threshold, we rebuilt the network and quantified the number of retained nodes, edges, and connected components relative to the original network. We also re-evaluated enrichment of putative effectors and predicted AMPs within structural clusters using the same settings as for the original network.

### Structural comparisons within *Z. passerinii* clusters and *Zymoseptoria* homologs

We investigated sequence conservation among proteins belonging to the same structural cluster by generating multiple sequence alignments (MSAs) using the L-INS-i algorithm implemented in MAFFT v7.525 [96]. Alignments were trimmed with trimAl v1.5.rev0 [97] using the “-gappyout” option and pairwise percent identity was computed from the MSAs using Biopython v1.78 [98]. To assess structural conservation, we used FoldMason [99] with default parameters to perform multiple structure alignments of proteins within a structural cluster. To quantify the variation between aligned residues, Euclidean distances were calculated using Cα atoms as reference points, as implemented in Biopython.

Sequence-based homologous relationships among *Z. passerinii* Zpa796 and other *Zymoseptoria* species (Supplementary Material 1: Table S12) were identified using OrthoFinder v2.2.7 [100] through an all-versus-all comparison with DIAMOND v0.9.24.125 [101]. Homologous proteins from *Z. passerinii* Zpa796 and *Z. tritici* IPO323 were aligned using TM-align. Protein structures from *Z. tritici* were based on the reference genome of the isolate IPO323 and retrieved from [60]. Distance maps for pairwise structural alignments were generated using Cα atoms to calculate residue-residue distances. For aligned protein pairs, residue-level hydrophobicity was calculated using the Kyte-Doolittle scale [102].

### Phylogenetic and selection analyses

To investigate the evolutionary relationships of proteins from selected structural clusters, we retrieved homologous proteins from other species based on the OrthoFinder results. Amino acid sequences were aligned with MAFFT L-INS-i and trimmed with trimAl. We determined the best-fit evolutionary model using ModelFinder, as implemented in IQ-TREE v2.3.6 [103], based on the Bayesian Information Criterion (BIC). Maximum-likelihood phylogenetic analyses were performed in IQ-TREE for each dataset with 10 independent runs and 1,000 ultrafast bootstrap replicates. The consensus trees were visualized using iTOL v7.5 [104].

To test for selection at the codon level, we analyzed the *Z. passerinii* Zpa796 proteins within selected structural clusters. Selection analyses were performed in HyPhy v2.5.64 [105] using two methods: MEME to detect episodic diversifying selection at individual sites and FEL to detect pervasive site-level selection. Detailed parameters used for alignments, trimming, phylogenetic inference, and codon-level selection are described in Supplementary Material 3.

### Comparison of physicochemical properties of proteins

The antimicrobial activity of proteins is also governed by physicochemical properties that influence their interactions with microbial membranes [43]. We quantified four properties from mature protein sequences using AMAPEC: net charge (at pH 7), hydrophobic moment (computed as the maximum helical amphipathicity), mean hydrophobicity, and surface hydrophobicity. Parameters used by AMAPEC to compute each of these four physicochemical properties are described in Supplementary Material 3. Additionally, we calculated charge density, defined as absolute net charge normalized by sequence length (net charge per residue). Statistical analyses were performed on proteins present in the structural similarity network, considering these five physicochemical properties plus mature protein length. The six properties were evaluated at two levels (functional groups of proteins in the network and structural clusters) in R v4.3.3 [106]. Differences among functional groups and structural clusters were assessed with the Kruskal-Wallis test (p-value < 0.05) followed by Dunn’s test for post hoc pairwise comparisons (p-value_adj_ < 0.05, using the Benjamini–Hochberg correction). Effect sizes for Kruskal-Wallis tests were calculated as epsilon-squared based on the H statistic. Pairwise post hoc effect sizes were calculated using Cliff’s delta. For cluster-level analyses, we restricted comparisons to clusters with at least three proteins to ensure statistical rigor.

## Supplementary Information


Supplementary Material 1.



Supplementary Material 2.



Supplementary Material 3.


## Data Availability

The datasets supporting the conclusions of this article are available in the Zenodo repository ( https://doi.org/10.5281/zenodo.16792728 ). The genome sequence and gene models used in this study for Z. passerinii isolate Zpa796 were retrieved from [34]. Code for the reproduction of the analyses in this paper is available across GitHub repositories: for comparative structural genomics ( https://github.com/thaisdalsasso/Structural_genomics_Zpa796 ) and protein annotation ( https://github.com/thaisdalsasso/Protein_annotation ).

## Abbreviations

AF2: AlphaFold2
AMPs: Antimicrobial proteins
pLDDT: Predicted local distance difference test
KP4: Killer toxin family 4
KP6: Killer toxin family 6
PDB: Protein Data Bank
pI: Isoelectric point
RMSD: Root mean square deviation
TM: Template modeling

## References

[1] Lo Presti L, Lanver D, Schweizer G, Tanaka S, Liang L, Tollot M, et al. Fungal effectors and plant susceptibility. Annu Rev Plant Biol. 2015;66:513–45. 10.1146/annurev-arplant-043014-114623. 25923844

[2] Snelders NC, Kettles GJ, Rudd JJ, Thomma BPHJ. Plant pathogen effector proteins as manipulators of host microbiomes? Mol Plant Pathol. 2018;19:257–9. 10.1111/mpp.12628. 29368817 PMC5817402

[3] Snelders NC, Rovenich H, Thomma BPHJ. Microbiota manipulation through the secretion of effector proteins is fundamental to the wealth of lifestyles in the fungal kingdom. FEMS Microbiol Rev. 2022;46:fuac022. 10.1093/femsre/fuac022. 35604874 PMC9438471

[4] Lovelace AH, Dorhmi S, Hulin MT, Li Y, Mansfield JW, Ma W. Effector identification in plant pathogens. Phytopathology. 2023;113:637–50. 10.1094/PHYTO-09-22-0337-KD. 37126080

[5] Sánchez-Vallet A, Fouché S, Fudal I, Hartmann FE, Soyer JL, Tellier A, et al. The genome biology of effector gene evolution in filamentous plant pathogens. Annu Rev Phytopathol. 2018;56:21–40. 10.1146/annurev-phyto-080516-035303. 29768136

[6] Seong K, Krasileva KV. Prediction of effector protein structures from fungal phytopathogens enables evolutionary analyses. Nat Microbiol. 2023;8:174–87. 10.1038/s41564-022-01287-6. 36604508 PMC9816061

[7] Li G, Newman M, Yu H, Rashidzade M, Martínez-Soto D, Caicedo A, et al. Fungal effectors: Past, present, and future. Curr Opin Microbiol. 2024;81:102526. 10.1016/j.mib.2024.102526. 39180827 PMC11442010

[8] Rocafort M, Fudal I, Mesarich CH. Apoplastic effector proteins of plant-associated fungi and oomycetes. Curr Opin Plant Biol. 2020;56:9–19. 10.1016/j.pbi.2020.02.004. 32247857

[9] de Jonge R, van Esse HP, Kombrink A, Shinya T, Desaki Y, Bours R, et al. Conserved Fungal LysM effector Ecp6 prevents chitin-triggered immunity in plants. Science. 2010;329:953–5. 10.1126/science.1190859. 20724636

[10] Cord-Landwehr S, Melcher RLJ, Kolkenbrock S, Moerschbacher BM. A chitin deacetylase from the endophytic fungus *Pestalotiopsis* sp. efficiently inactivates the elicitor activity of chitin oligomers in rice cells. Sci Rep. 2016;6:38018. 10.1038/srep38018. 27901067 PMC5128826

[11] Hemetsberger C, Herrberger C, Zechmann B, Hillmer M, Doehlemann G. The *Ustilago maydis* effector Pep1 suppresses plant immunity by inhibition of host peroxidase activity. PLoS Pathog. 2012;8:e1002684. 10.1371/journal.ppat.1002684. 22589719 PMC3349748

[12] Djamei A, Schipper K, Rabe F, Ghosh A, Vincon V, Kahnt J, et al. Metabolic priming by a secreted fungal effector. Nature. 2011;478:395–8. 10.1038/nature10454. 21976020

[13] Shabab M, Shindo T, Gu C, Kaschani F, Pansuriya T, Chintha R, et al. Fungal effector protein AVR2 targets diversifying defense-related cys proteases of tomato. Plant Cell. 2008;20:1169–83. 10.1105/tpc.107.056325. 18451324 PMC2390736

[14] Sung YC, Outram MA, Breen S, Wang C, Dagvadorj B, Winterberg B, et al. PR1-mediated defence via C-terminal peptide release is targeted by a fungal pathogen effector. New Phytol. 2021;229:3467–80. 10.1111/nph.17128. 33277705

[15] Snelders NC, Petti GC, Van Den Berg GCM, Seidl MF, Thomma BPHJ, Lindow SE. An ancient antimicrobial protein co-opted by a fungal plant pathogen for in planta mycobiome manipulation. Proc Natl Acad Sci U S A. 2021;118:e2110968118. 10.1073/pnas.2110968118. 34853168 PMC8670511

[16] Flores-Nunez VM, Stukenbrock EH. The impact of filamentous plant pathogens on the host microbiota. BMC Biol. 2024;22:175. 10.1186/s12915-024-01965-3. 39148076 PMC11328434

[17] Jones DA, Bertazzoni S, Turo CJ, Syme RA, Hane JK. Bioinformatic prediction of plant–pathogenicity effector proteins of fungi. Curr Opin Microbiol. 2018;46:43–9. 10.1016/j.mib.2018.01.017. 29462764

[18] Kumar R, Acharya V. Effector protein structures: A tale of evolutionary relationship. Trends Plant Sci. 2023;28:746–8. 10.1016/j.tplants.2023.04.010. 37127498

[19] Sperschneider J, Dodds PN. EffectorP 3.0: Prediction of apoplastic and cytoplasmic effectors in fungi and oomycetes. Mol Plant Microbe Interact. 2022;35:146–56. 10.1094/MPMI-08-21-0201-R. 34698534

[20] Seong K, Krasileva KV. Computational structural genomics unravels common folds and novel families in the secretome of fungal phytopathogen *Magnaporthe oryzae*. Mol Plant Microbe Interact. 2021;34:1267–80. 10.1094/MPMI-03-21-0071-R. 34415195 PMC9447291

[21] Derbyshire MC, Raffaele S. Surface frustration re-patterning underlies the structural landscape and evolvability of fungal orphan candidate effectors. Nat Commun. 2023;14:5244. 10.1038/s41467-023-40949-9. 37640704 PMC10462633

[22] Jones DAB, Raffaele S. Phytopathogen effector biology in the burgeoning AI era. Annu Rev Phytopathol. 2025;63:63–88. 10.1146/annurev-phyto-121823. 40541232

[23] Outram MA, Figueroa M, Sperschneider J, Williams SJ, Dodds PN. Seeing is believing: Exploiting advances in structural biology to understand and engineer plant immunity. Curr Opin Plant Biol. Volume 67. Elsevier Ltd; 2022. p. 102210. 10.1016/j.pbi.2022.102210. 35461025

[24] Jumper J, Evans R, Pritzel A, Green T, Figurnov M, Ronneberger O, et al. Highly accurate protein structure prediction with AlphaFold. Nature. 2021;596:583–9. 10.1038/s41586-021-03819-2. 34265844 PMC8371605

[25] Lin Z, Akin H, Rao R, Hie B, Zhu Z, Lu W, et al. Evolutionary-scale prediction of atomic-level protein structure with a language model. Science. 2023;379:1123–30. 10.1126/science.ade2574. 36927031

[26] Yu DS, Outram MA, Smith A, McCombe CL, Khambalkar PB, Rima SA, et al. The structural repertoire of *Fusarium oxysporum* f. sp. *lycopersici* effectors revealed by experimental and computational studies. eLife. 2024;12:RP89280. 10.7554/eLife.89280.2. 38411527 PMC10942635

[27] Mukhopadhyay S, Javed MA, Wu J, Pérez-López E. Structure-guided secretome analysis of gall-forming microbes offers insights into effector diversity and evolution. eLife. 2025;14:RP105185. 10.7554/eLife.105185.1. 41055214 PMC12503489

[28] Rocafort M, Bowen JK, Hassing B, Cox MP, McGreal B, de la Rosa S, et al. The *Venturia inaequalis* effector repertoire is dominated by expanded families with predicted structural similarity, but unrelated sequence, to avirulence proteins from other plant-pathogenic fungi. BMC Biol. 2022;20:246. 10.1186/s12915-022-01442-9. 36329441 PMC9632046

[29] Feurtey A, Lorrain C, Croll D, Eschenbrenner C, Freitag M, Habig M, et al. Genome compartmentalization predates species divergence in the plant pathogen genus *Zymoseptoria*. BMC Genomics. 2020;21:588. 10.1186/s12864-020-06871-w. 32842972 PMC7448473

[30] Torriani SFF, Melichar JPE, Mills C, Pain N, Sierotzki H, Courbot M. *Zymoseptoria tritici*: A major threat to wheat production, integrated approaches to control. Fungal Genet Biol. 2015;79:8–12. 10.1016/j.fgb.2015.04.010. 26092783

[31] Feurtey A, Lorrain C, McDonald MC, Milgate A, Solomon PS, Warren R, et al. A thousand-genome panel retraces the global spread and adaptation of a major fungal crop pathogen. Nat Commun. 2023;14:1059. 10.1038/s41467-023-36674-y. 36828814 PMC9958100

[32] Toubia-Rahme H, Steffenson BJ. Sources of resistance to septoria speckled leaf blotch caused by *Septoria passerinii* in barley. Can J Plant Pathol. 2004;26:358–64. 10.1080/07060660409507153.

[33] Rojas-Barrera IC, Fagundes WC, Stukenbrock EH. Species of *Zymoseptoria* (Dothideomycetes) as a model system to study plant pathogen genome evolution. In: Scott B, Mesarich C, editors. Plant Relationships: Fungal-Plant Interactions. Cham: Springer International Publishing; 2023. pp. 349–70. 10.1007/978-3-031-16503-0_15.

[34] Rojas-Barrera IC, Flores‐Núñez VM, Haueisen J, Alizadeh A, Salimi F, Stukenbrock EH. Evolution of sympatric host‐specialized lineages of the fungal plant pathogen *Zymoseptoria passerinii* in natural ecosystems. New Phytol. 2025;245:1673–87. 10.1111/nph.20340. 39686531 PMC11754930

[35] Stukenbrock EH, Banke S, Javan-Nikkhah M, McDonald BA. Origin and domestication of the fungal wheat pathogen *Mycosphaerella graminicola* via sympatric speciation. Mol Biol Evol. 2007;24:398–411. 10.1093/molbev/msl169. 17095534

[36] Flores-Núñez VM, Dal’Sasso TCS, Hansen M, Reinhardt G, Braun S, Stukenbrock EH. Host-specific fungal plant pathogens exhibit distinct interactions with the leaf microbiota of wild grasses. Philosophical Trans B. 2026;381. 10.1098/rstb.2025.0112. 42206330

[37] Berman HM, Westbrook J, Feng Z, Gilliland G, Bhat TN, Weissig H, et al. The Protein Data Bank. Nucleic Acids Res. 2000;28:235–42. 10.1093/nar/28.1.235. 10592235 PMC102472

[38] Mesny F, Wolf V, López-Moral A, Kraege A, Punt W, Liu S, et al. Plant-associated fungi co-opt ancient antimicrobials for host manipulation. Sci Adv. 2026;12:1406. 10.1126/sciadv.aec1406. PMC13127573 42054466

[39] Knudsen M, Wiuf C. The CATH database. Hum Genomics. 2010;4:207–12. 10.1186/1479-7364-4-3-207. 20368142 PMC3525972

[40] Fox NK, Brenner SE, Chandonia JM, SCOPe. Structural Classification of Proteins - Extended, integrating SCOP and ASTRAL data and classification of new structures. Nucleic Acids Res. 2014;42:D304–9. 10.1093/nar/gkt1240. 24304899 PMC3965108

[41] Cheng H, Schaeffer RD, Liao Y, Kinch LN, Pei J, Shi S, et al. ECOD: An Evolutionary Classification of Protein Domains. PLoS Comput Biol. 2014;10:e1003926. 10.1371/journal.pcbi.1003926. 25474468 PMC4256011

[42] Malik AJ, Poole AM, Allison JR. Structural phylogenetics with confidence. Mol Biol Evol. 2020;37:2711–26. 10.1093/molbev/msaa100. 32302382 PMC7475046

[43] Yeaman MR, Yount NY. Mechanisms of antimicrobial peptide action and resistance. Pharmacol Rev. 2003;55:27–55. 10.1124/pr.55.1.2. 12615953

[44] Arai M, Suetaka S, Ooka K. Dynamics and interactions of intrinsically disordered proteins. Curr Opin Struct Biol. 2024;84:102734. 10.1016/j.sbi.2023.102734. 38039868

[45] Ali H, Thynne E, Ellis T, Booth C, Bates R, Hope MS, et al. Molecular mimicry of plant cell-surface immune receptors by fungal secreted leucine-rich repeat proteins. bioRxiv. 2025. 10.1101/2025.08.22.671746.41446184

[46] Pirc K, Clifton LA, Yilmaz N, Saltalamacchia A, Mally M, Snoj T, et al. An oomycete NLP cytolysin forms transient small pores in lipid membranes. Sci Adv. 2022;8:9406. 10.1126/sciadv.abj9406. PMC8916740 35275729

[47] Chruszcz M, Chapman MD, Osinski T, Solberg R, Demas M, Porebski PJ, et al. *Alternaria alternata* allergen Alt a 1: A unique β-barrel protein dimer found exclusively in fungi. J Allergy Clin Immunol. 2012;130:241–e2479. 10.1016/j.jaci.2012.03.047. 22664167 PMC3391610

[48] Sabotič J, Renko M, Kos J. β-Trefoil protease inhibitors unique to higher fungi. Acta Chim Slov. 2019;66:28–36. 10.17344/acsi.2018.4465. 33855482

[49] Zhang Y, Liang Y, Dong Y, Gao Y, Yang X, Yuan J, et al. The *Magnaporthe oryzae* Alt A 1-like protein MoHrip1 binds to the plant plasma membrane. Biochem Biophys Res Commun. 2017;492:55–60. 10.1016/j.bbrc.2017.08.039. 28807829

[50] Zhang Y, Gao Y, Liang Y, Dong Y, Yang X, Qiu D. *Verticillium dahliae* PevD1, an Alt a 1-like protein, targets cotton PR5-like protein and promotes fungal infection. J Exp Bot. 2019;70:613–26. 10.1093/jxb/ery351. 30295911 PMC6322577

[51] Haueisen J, Möller M, Seybold H, Small C, Wilkens M, Jahneke L, et al. Comparative analyses of compatible and incompatible host-pathogen interactions provide insight into divergent host specialization of closely related pathogens. Mol Plant Microbe Interact. 2025;38:235–51. 10.1094/MPMI-10-24-0133-FI. 39999443

[52] Varrot A, Basheer SM, Imberty A. Fungal lectins: Structure, function and potential applications. Curr Opin Struct Biol. 2013;23:678–85. 10.1016/j.sbi.2013.07.007. 23920351

[53] Žurga S, Pohleven J, Kos J, Sabotič J. β-Trefoil structure enables interactions between lectins and protease inhibitors that regulate their biological functions. J Biochem. 2015;158:83–90. 10.1093/jb/mvv025. 25742738

[54] Renko M, Zupan T, Plaza DF, Schmieder SS, Nanut MP, Kos J, et al. Cocaprins, β-trefoil fold inhibitors of cysteine and aspartic proteases from *Coprinopsis cinerea*. Int J Mol Sci. 2022;23:4916. 10.3390/ijms23094916. 35563308 PMC9104457

[55] Vicente I, Quaratiello G, Baroncelli R, Vannacci G, Sarrocco S. Insights on KP4 Killer Toxin-like Proteins of *Fusarium* species in interspecific interactions. J Fungi. 2022;8:968. 10.3390/jof8090968. PMC9506348 36135693

[56] Brown DW. The KP4 killer protein gene family. Curr Genet. 2011;57:51–62. 10.1007/s00294-010-0326-y. 21116630

[57] Gage MJ, Bruenn J, Fischer M, Sanders D, Smith TJ. KP4 fungal toxin inhibits growth in *Ustilago maydis* by blocking calcium uptake. Mol Microbiol. 2001;41:775–85. 10.1046/j.1365-2958.2001.02554.x. 11532143

[58] Lazar N, Mesarich CH, Petit-Houdenot Y, Talbi N, de la Sierra-Gallay IL, Zélie E, et al. A new family of structurally conserved fungal effectors displays epistatic interactions with plant resistance proteins. PLoS Pathog. 2022;18:e1010664. 10.1371/journal.ppat.1010664. 35793393 PMC9292093

[59] Mesarich CH, Ökmen B, Rovenich H, Griffiths SA, Wang C, Jashni MK, et al. Specific hypersensitive response-associated recognition of new apoplastic effectors from *Cladosporium fulvum* in wild tomato. Mol Plant Microbe Interact. 2018;31:145–62. 10.1094/MPMI-05-17-0114-FI. 29144204

[60] Thynne E, Ali H, Seong K, Abukhalaf M, Guerreiro MA, Flores-Nunez VM, et al. An array of Zymoseptoria tritici effectors suppress plant immune responses. Mol Plant Pathol. 2024;25:e13500. 10.1111/mpp.13500. 39394693 PMC11470090

[61] de Guillen K, Mammri L, Gracy J, Padilla A, Barthe P, Hoh F, et al. *Zymoseptoria tritici* effectors structurally related to Killer Proteins UmV-KP4 and UmV-KP6 inhibit fungal growth, and define extended protein families in fungi. Mol Plant Pathol. 2025;26:e70141. 10.1111/mpp.70141. 40864528 PMC12382754

[62] Mesny F, Bauer M, Zhu J, Thomma BPHJ. Meddling with the microbiota: Fungal tricks to infect plant hosts. Curr Opin Plant Biol. 2024;82:102622. 10.1016/j.pbi.2024.102622. 39241281

[63] Pachinger L, Stukenbrock EH. Antimicrobial effectors of plant-associated fungi: multipurpose proteins with fast-evolving surfaces and structurally conserved cores. Curr Opin Microbiol. 2026;91. 10.1016/j.mib.2026.102744. 41903508

[64] Chakrabarti P, Chakravarty D. Intrinsically disordered proteins/regions and insight into their biomolecular interactions. Biophys Chem. 2022;283:106769. 10.1016/j.bpc.2022.106769. 35139468

[65] Tempra C, La Rosa C, Lolicato F. The role of alpha-helix on the structure-targeting drug design of amyloidogenic proteins. Chem Phys Lipids. 2021;236:105061. 10.1016/j.chemphyslip.2021.105061. 33610597

[66] Van Der Weerden NL, Bleackley MR, Anderson MA. Properties and mechanisms of action of naturally occurring antifungal peptides. Cell Mol Life Sci. 2013;70:3545–70. 10.1007/s00018-013-1260-1. 23381653 PMC11114075

[67] Sato H, Feix JB. Peptide-membrane interactions and mechanisms of membrane destruction by amphipathic α-helical antimicrobial peptides. Biochim Biophys Acta Biomembr. 2006;1758:1245–56. 10.1016/j.bbamem.2006.02.021. 16697975

[68] Yaeno T, Li H, Chaparro-Garcia A, Schornack S, Koshiba S, Watanabe S, et al. Phosphatidylinositol monophosphate-binding interface in the oomycete RXLR effector AVR3a is required for its stability in host cells to modulate plant immunity. Proc Natl Acad Sci U S A. 2011;108:14682–7. 10.1073/pnas.1106002108. 21821794 PMC3167543

[69] Blondeau K, Blaise F, Graille M, Kale SD, Linglin J, Ollivier B, et al. Crystal structure of the effector AvrLm4-7 of *Leptosphaeria maculans* reveals insights into its translocation into plant cells and recognition by resistance proteins. Plant J. 2015;83:610–24. 10.1111/tpj.12913. 26082394

[70] Rocha VD, Dal’Sasso TCS, Dal-Bianco M, Oliveira LO. Genome-wide survey and evolutionary history of the pectin methylesterase (PME) gene family in the Dothideomycetes class of fungi. Fungal Genet Biol. 2023;169:103841. 10.1016/j.fgb.2023.103841. 37797717

[71] Dal’Sasso TCS, Rocha VD, Rody HVS, Costa MDBL, Oliveira LO. The Necrosis- and Ethylene-inducing peptide 1-like protein (NLP) gene family of the plant pathogen *Corynespora cassiicola*. Curr Genet. 2022;68:645–59. 10.1007/s00294-022-01252-0. 36098767

[72] Papaleo E, Saladino G, Lambrughi M, Lindorff-Larsen K, Gervasio FL, Nussinov R. The role of protein loops and linkers in conformational dynamics and allostery. Chem Rev. 2016;116:6391–423. 10.1021/acs.chemrev.5b00623. 26889708

[73] Shehu A, Kavraki LE. Modeling structures and motions of loops in protein molecules. Entropy. 2012;14:252–90. 10.3390/e14020252.

[74] Krutyhołowa R, Reinhardt-Tews A, Chramiec-Głąbik A, Breunig KD, Glatt S. Fungal Kti12 proteins display unusual linker regions and unique ATPase p-loops. Curr Genet. 2020;66:823–33. 10.1007/s00294-020-01070-2. 32236652 PMC7363723

[75] Weiland P, Dempwolff F, Steinchen W, Freibert SA, Tian H, Glatter T, et al. Structural and functional analysis of the cerato-platanin-like protein Cpl1 suggests diverging functions in smut fungi. Mol Plant Pathol. 2023;24:768–87. 10.1111/mpp.13349. 37171083 PMC10257043

[76] Kozome D, Sljoka A, Laurino P. Remote loop evolution reveals a complex biological function for chitinase enzymes beyond the active site. Nat Commun. 2024;15:3227. 10.1038/s41467-024-47588-8. 38622119 PMC11018821

[77] Felle HH, Herrmann A, Hanstein S, Hückelhoven R, Kogel K-H. Apoplastic pH signaling in barley leaves attacked by the powdery mildew fungus *Blumeria graminis* f. sp. *hordei*. Mol Plant Microbe Interact. 2004;17:118–23. 10.1094/MPMI.2004.17.1.118. 14714875

[78] Aleandri MP, Magro P, Chilosi G. Modulation of host pH during the wheat-*Fusarium culmorum* interaction and its influence on the production and activity of pectolytic enzymes. Plant Pathol. 2007;56:517–25. 10.1111/j.1365-3059.2007.01574.x.

[79] Allen A, Chatt E, Smith TJ. The atomic structure of the virally encoded antifungal protein, KP6. J Mol Biol. 2013;425:609–21. 10.1016/j.jmb.2012.11.033. 23219466

[80] Jones P, Binns D, Chang HY, Fraser M, Li W, McAnulla C, et al. InterProScan 5: Genome-scale protein function classification. Bioinformatics. 2014;30:1236–40. 10.1093/bioinformatics/btu031. 24451626 PMC3998142

[81] Cantalapiedra CP, Hern̗andez-Plaza A, Letunic I, Bork P, Huerta-Cepas J. eggNOG-mapper v2: Functional annotation, orthology assignments, and domain prediction at the metagenomic scale. Mol Biol Evol. 2021;38:5825–9. 10.1093/molbev/msab293. 34597405 PMC8662613

[82] Blin K, Shaw S, Augustijn HE, Reitz ZL, Biermann F, Alanjary M, et al. antiSMASH 7.0: New and improved predictions for detection, regulation, chemical structures and visualisation. Nucleic Acids Res. 2023;51:W46–50. 10.1093/nar/gkad344. 37140036 PMC10320115

[83] Teufel F, Almagro Armenteros JJ, Johansen AR, Gíslason MH, Pihl SI, Tsirigos KD, et al. SignalP 6.0 predicts all five types of signal peptides using protein language models. Nat Biotechnol. 2022;40:1023–5. 10.1038/s41587-021-01156-3. 34980915 PMC9287161

[84] Käll L, Krogh A, Sonnhammer ELL. Advantages of combined transmembrane topology and signal peptide prediction-the Phobius web server. Nucleic Acids Res. 2007;35:W429–32. 10.1093/nar/gkm256. 17483518 PMC1933244

[85] Krogh A, Larsson B, Von Heijne G, Sonnhammer ELL. Predicting transmembrane protein topology with a hidden Markov model: Application to complete genomes. J Mol Biol. 2001;305:567–80. 10.1006/jmbi.2000.4315. 11152613

[86] Emanuelsson O, Brunak S, von Heijne G, Nielsen H. Locating proteins in the cell using TargetP, SignalP and related tools. Nat Protoc. 2007;2:953–71. 10.1038/nprot.2007.131. 17446895

[87] Thumuluri V, Almagro Armenteros JJ, Johansen AR, Nielsen H, Winther O. DeepLoc 2.0: Multi-label subcellular localization prediction using protein language models. Nucleic Acids Res. 2022;50:W228–34. 10.1093/nar/gkac278. 35489069 PMC9252801

[88] Walsh I, Martin AJM, Di Domenico T, Tosatto SCE, Espritz. Accurate and fast prediction of protein disorder. Bioinformatics. 2012;28:503–9. 10.1093/bioinformatics/btr682. 22190692

[89] Zhang Y, Skolnick J. TM-align: A protein structure alignment algorithm based on the TM-score. Nucleic Acids Res. 2005;33:2302–9. 10.1093/nar/gki524. 15849316 PMC1084323

[90] van Kempen M, Kim SS, Tumescheit C, Mirdita M, Lee J, Gilchrist CLM, et al. Fast and accurate protein structure search with Foldseek. Nat Biotechnol. 2024;42:243–6. 10.1038/s41587-023-01773-0. 37156916 PMC10869269

[91] Varadi M, Anyango S, Deshpande M, Nair S, Natassia C, Yordanova G, et al. AlphaFold Protein Structure Database: Massively expanding the structural coverage of protein-sequence space with high-accuracy models. Nucleic Acids Res. 2022;50:D439–44. 10.1093/nar/gkab1061. 34791371 PMC8728224

[92] Xu J, Zhang Y. How significant is a protein structure similarity with TM-score = 0.5? Bioinformatics. 2010;26:889–95. 10.1093/bioinformatics/btq066. 20164152 PMC2913670

[93] Hagberg A, Schult D, Swart PJ. Exploring network structure, dynamics, and function using NetworkX. In: Proceedings of the 7th Python Science Conference (SciPy 2008). Pasadena; 2008:11–5. 10.25080/TCWV9851.

[94] Shannon P, Markiel A, Ozier O, Baliga NS, Wang JT, Ramage D, et al. Cytoscape: A software Environment for integrated models of biomolecular interaction networks. Genome Res. 2003;13:2498–504. 10.1101/gr.1239303. 14597658 PMC403769

[95] Virtanen P, Gommers R, Oliphant TE, Haberland M, Reddy T, Cournapeau D, et al. SciPy 1.0: Fundamental algorithms for scientific computing in Python. Nat Methods Nat Res. 2020;17:261–72. 10.1038/s41592-019-0686-2. PMC7056644 32015543

[96] Katoh K, Standley DM. MAFFT multiple sequence alignment software version 7: Improvements in performance and usability. Mol Biol Evol. 2013;30:772–80. 10.1093/molbev/mst010. 23329690 PMC3603318

[97] Capella-Gutiérrez S, Silla-Martínez JM, Gabaldón T, trimAl. A tool for automated alignment trimming in large-scale phylogenetic analyses. Bioinformatics. 2009;25:1972–3. 10.1093/bioinformatics/btp348. 19505945 PMC2712344

[98] Cock PJA, Antao T, Chang JT, Chapman BA, Cox CJ, Dalke A, et al. Biopython: Freely available Python tools for computational molecular biology and bioinformatics. Bioinformatics. 2009;25:1422–3. 10.1093/bioinformatics/btp163. 19304878 PMC2682512

[99] Gilchrist CLM, Mirdita M, Steinegger M. Multiple protein structure alignment at scale with FoldMason. bioRxiv. 2024. 10.1101/2024.08.01.606130. 41610233

[100] Emms DM, Kelly S, OrthoFinder. Solving fundamental biases in whole genome comparisons dramatically improves orthogroup inference accuracy. Genome Biol. 2015;16:1–14. 10.1186/s13059-015-0721-2. 26243257 PMC4531804

[101] Buchfink B, Xie C, Huson DH. Fast and sensitive protein alignment using DIAMOND. Nat Methods. 2014;12:59–60. 10.1038/nmeth.3176. 25402007

[102] Kyte J, Doolittle RF. A simple method for displaying the hydropathic character of a protein. J Mol Biol. 1982;157:105–32. 10.1016/0022-2836(82)90515-0. 7108955

[103] Nguyen LT, Schmidt HA, Von Haeseler A, Minh BQ. IQ-TREE: A fast and effective stochastic algorithm for estimating maximum-likelihood phylogenies. Mol Biol Evol. 2015;32:268–74. 10.1093/molbev/msu300. 25371430 PMC4271533

[104] Letunic I, Bork P. Interactive Tree of Life (iTOL) v6: Recent updates to the phylogenetic tree display and annotation tool. Nucleic Acids Res. 2024;52:W78–82. 10.1093/nar/gkae268. 38613393 PMC11223838

[105] Kosakovsky Pond SL, Poon AFY, Velazquez R, Weaver S, Hepler NL, Murrell B, et al. HyPhy 2.5 - A Customizable Platform for Evolutionary Hypothesis Testing Using Phylogenies. Mol Biol Evol. 2020;37:295–9. 10.1093/molbev/msz197. 31504749 PMC8204705

[106] R Core Team. R: A language and environment for statistical computing. https://www.R-project.org/. Accessed 11 Aug 2025.

